# Prognostic value of lymph node micrometastasis in esophageal cancer: A systematic review and meta-analysis

**DOI:** 10.3389/fonc.2022.1025855

**Published:** 2023-01-04

**Authors:** Jing Yang, Qianqian Liu, Yuping Bai, Haitong Zhao, Tingting He, Ziru Zhao, Min Huang, Mengyuan Jiang, Rui Zhang, Min Zhang

**Affiliations:** ^1^ School of Basic Medicine, Gansu University of Traditional Chinese Medicine, Lanzhou, Gansu, China; ^2^ Department of Pathology, Gansu Provincial Hospital, Lanzhou, Gansu, China; ^3^ Department of Pathology, The 940th Hospital of Joint Logistics Support Force of Chinese People´s Liberation Army, Lanzhou, China; ^4^ The Department of Pathology, Hainan Provincial Hospital, Haikou, Hainan, China; ^5^ Evidence Based Social Science Research Center, School of Public Health, Lanzhou University, Lanzhou, China

**Keywords:** esophageal cancer, lymph node micrometastasis, prognosis, meta-analysis, systematic review

## Abstract

**Objective:**

Whether lymph node micrometastasis (LNM) increases the risk in esophageal cancer patients remains controversial. We conducted a systematic review and meta-analysis to explore the prognosis value of LNM in esophageal cancer patients.

**Methods:**

Two reviewers independently searched electronic databases, including PubMed, Embase, and the Cochrane Library, for eligible citations until February 2022. We calculated pooled estimates of the hazards ratio with a random-effects model. The certainty of evidence was determined by the Grade of Recommendations Assessment, Development, and Evaluation (GRADE) method. A sensitivity analysis was performed to assess the stability. Publication bias was assessed using funnel plots and Egger’s test. We also performed subgroup analysis to explore the source of heterogeneity.

**Results:**

A total of 16 studies, with 1,652 patients, were included. The overall survival (OS) was significantly increased with LNM negativity compared with LNM positivity (HR 1.95; 95% CI, 1.53–2.49; *P <* 0.001; *I^2^* = 0.0%, *P* = 0.930; certainty of evidence: low). Relapse-free survival (RFS) was significantly increased with LNM negativity compared with LNM positivity (HR 3.39; 95% CI, 1.87–6.16; *P <* 0.001; *I^2^* = 50.18%, *P* = 0.060; certainty of evidence: moderate). No significant difference was observed in recurrence between the two groups (certainty of evidence: low). Sensitivity analysis revealed a stable trend. In addition, the funnel plot and Egger’s test did not show significant publication bias.

**Conclusion:**

LNM positivity worsens the prognosis in esophageal cancer, and the evidence for RFS is moderate. Future relevant high-quality studies are warranted to validate our results further and provide a reference for guidelines.

**Systematic review registration:**

https://www.crd.york.ac.uk/prospero, identifier (CRD42022321768).

## 1 Introduction

The occurrence of esophageal cancer has increased in the Western world over the past few years and is expected to further rise (1, 2). Despite improvements in diagnostic methods and treatment, many patients are at the risk of recurrence post-surgery. Recurrence is likely to be associated with lymph node involvement as this is the strongest prognostic factor in esophageal cancer, with a 5-year survival rate in patients with pN3 ranging from 2% to 17% and that in patients with pN0 (no lymph node metastasis) being up to 83% (3). Lymph node micrometastasis (LNM) can be detected in the pN0 stage. In the presence of LNM, the 5-year survival rate for patients with esophageal cancer varies from less than 1% to 30% (4). Therefore, LNM may be a good survival predictor.

LNM is challenging to identify with certainty by routine Hematoxylin and Eosin (HE) staining. However, immunohistochemical (IHC) staining for cytokeratin can highlight small tumor cells, making them more easily detectable. A previous study (5) has reported that patients with LNM had significantly lower disease-free survival rates than those with negative lymph node metastasis in esophageal cancer. Another study (6) has reported that patients with LNM have a higher local recurrence rate than those without LNM. Another study (7) performed multivariate cox regression analysis, which showed that LNM was an independent prognostic factor for 5-year relapse-free survival (RFS) rate; however, no statistical differences were found in the 5-year overall survival (OS) rate between patients with LNM and those without LNM. It has been reported (8) that patients with LNM have significantly lower disease-free survival rates, indicating a worse prognosis. Although LNM has been evaluated in esophageal cancer, it is not included in TNM staging of esophageal cancer because there are no vital pieces of evidence indicating that LNM has a negative prognostic impact on esophageal cancer (9).

Nonetheless, Union for International Cancer Control (UICC) and American Joint Committee on Cancer **(**AJCC) are watchful toward LNM (10, 11). Moreover, there is no meta-analysis quantifying the value of LNM in the prognosis of esophageal cancer. Therefore, we have performed this meta-analysis to assess the prognostic value of LNM in patients with esophageal cancer.

## 2 Methods

### 2.1 Data sources and search strategy

This meta-analysis was reported following the Preferred Reporting Items for Systematic Reviews and Meta-Analyses (PRISMA) Statement (12). The study has been registered at the International Prospective Register of Systematic Reviews (PROSPERO) (CRD42022321768) (13).

We searched for eligible studies in the electronic databases PubMed, Embase, and the Cochrane Library up to February 2022. We used the following combined text and MeSH terms: “Esophageal Neoplasms” and “Neoplasm Micrometastasis”. The complete literature search strategy for PubMed is provided in **Supplementary Appendices 1**. We also conducted a manual search using the reference lists of critical articles published in English.

### 2.2 Study selection and data extraction

The articles included in the analysis were selected based on the following eligibility criteria: (1) all patients were diagnosed with esophageal cancer and (2) studies included information on the prognostic value of LNM in esophageal cancer. Articles were excluded from the analysis if (1) they were not presented in English or Chinese; (2) they were review articles, meta-analyses, or conference abstracts; and (3) they did not include the available data.

Two independent investigators (JY and QQL) reviewed all relevant and eligible literature using standardized data-extraction forms. Disagreements were solved by consulting with a third investigator (MZ). The following data from each selected article: author names, year of publication, country/region, study design, total number of participants, age, sex, clinical outcomes, effect size with 95% CI, follow-up duration, and drop-out percentage were extracted.

### 2.3 Assessment of study quality

To assess the quality of each included study, two authors independently assessed the risk of bias using Quality In Prognosis Studies (QUIPS) tool (14). The studies were finally evaluated as “high risk of bias,” “moderate risk of bias,” and “low risk of bias”. The QUIPS tool included six crucial areas to evaluate validity and bias in studies of prognostic factors, including participation, attrition, measurement of prognostic factors, outcomes, confounding factors, statistical analysis, and reporting.

### 2.4 Assessment of quality of evidence

The certainty of evidence was determined following the Grade of Recommendations Assessment, Development and Evaluation (GRADE) method (15, 16). The assessment of evidence quality was based on five aspects: limitations, inconsistencies, indirectness, inaccuracies, and publication bias. The evidence quality of each outcome was rated as “high,” “moderate,” “low,” and “very low”.

### 2.5 Study outcomes

We assessed the effect of the prognostic value of LNM in esophageal cancer on three outcomes: OS, RFS, and recurrence. In five of the included studies (5, 8, 17–19), disease-free survival was regarded as RFS because they have the same definition.

### 2.6 Statistical analyses

All statistical analyses were performed using Stata (version 16.0). We calculated pooled estimates of the hazards ratio (HR) and odds ratio (OR) with a random-effects model (Dersimonian-Laird method). In the included articles (6, 20, 21), the HR of OS was transformed by a survival curve (22). The *I^2^* statistic and *P*-value of Cochrane’s Q test were used to assess the heterogeneity of effects, with I^2^ = 25%–50% indicating mild, 50%–75% indicating moderate, and >75% indicating severe heterogeneity (23, 24).

We specified subgroups to explore the source of heterogeneity. Several subgroups were also analyzed, including study design, which was divided into retrospective, prospective, and RCT; region, which was divided into Asia and non-Asia; antibody type, which was divided into Ber-EP4 and AE1/AE3; follow-up duration, which was divided into less than five years and more than five years; tumor type, which was divided into squamous cell carcinoma (SCC) and esophageal adenocarcinoma (EAC); pN status, which was divided into pN+ (studies including LNM in pN+ patients) and pN0 (studies restricted to LNM in pN0 patients); single-center/multicenter studies; and univariate/multivariate analysis.

A *P*-value < 0.05 indicated statistical significance. In addition, we assessed asymmetry using funnel plots and Egger’s test and defined significant publication bias if *P* value is <0.05. Finally, we conducted sensitivity analyses to evaluate the stability of the results. Sensitivity analysis was conducted for all studies except three wherein the HRs were transformed by a survival curve to verify the stability of our results (6, 20, 21).

## 3 Results

### 3.1 Study selection and baseline characteristics

**Figure 1:**
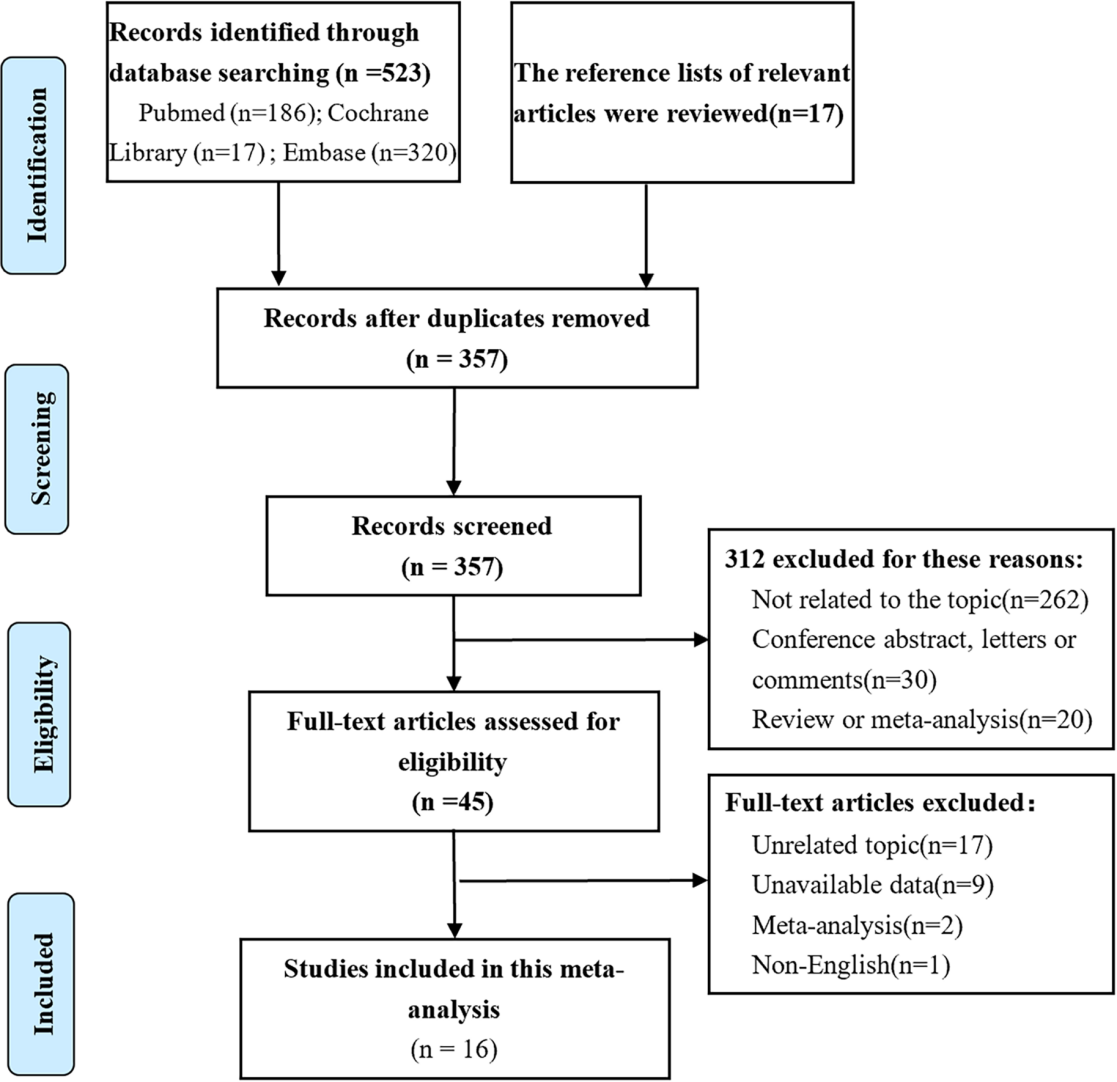
PRISMA flow diagram outlining the literature search process.

Of the 540 studies identified in our analysis, and 495 abstracts were retrieved and reviewed for possible inclusion after removing duplicates. Subsequently, 45 full-text manuscripts were assessed for eligibility, from which 29 did not meet the inclusion criteria and were excluded. Accordingly, only 16 studies including 1,652 patients were included (5–7, 17–21, 25–32) (**Figure 1**). The study characteristics and baseline demographics are shown (**Table 1**). The majority of studies were based in Asia. The study designs were randomized, retrospective, or prospective studies. LNM evaluation was performed *via* IHC staining with different antibody types that were classified as AE1/AE3 and Ber-EP4. Follow-up durations ranged from 1 month to 174 months. The basic features of esophageal cancer are shown in **Supplementary Appendices 2**.

**Table 1:** Study characteristics and baseline demographics.

First author	Year	Country	Study design	S/M	AgeMean ± SD/range, years	Patients, nP/N	Male, nP/N	LNs, n	Average LNs, n	Definition of LNM	Antibody types	Median follow-up, (range), month	Drop-out (n, %)
Izbicki,J.R. (25).	1997	Germany	POS	S	57.0(34.0-76.0)	42/26	33/21	1308	NA	conventional histopathological negative and IHC positive	Ber-EP4	21.0(2.0-51.0)	5, 7.4
Komukai,S., (26)	2000	Japan	POS	S	60.8 ± 7.3	14/23	12/20	2845	75(38-127)	pN0 and IHC positive	AE1/AE3	60.0	NA
Matsumoto,M., (27)	2000	Japan	POS	S	64.1(37.0-85.0)	33/26	27/24	2714	46(14-103) *	pN0 and IHC positive	AE1/AE3	60.0	0
Sato,F., (28)	2001	Japan	ROS	S	64.7 ± 9.0	20/30	15/21	1840	37(6-136)	pN0 and IHC positive	AE1/AE3	67.0(7.0-136.0)	0
Doki,Y., (20)	2002	Japan	ROS	M	NA	11/30	NA	2168	53	pN0 and IHC positive	AE1/AE3	120.0	0
Nakamura,T., (29)	2002	Japan	ROS	S	62.5(41.0-76.0)	14/39	NA	2511	49	pN0 and IHC positive	AE1/AE3	53.5(2.0-92.0)	0
Xiao,X.W. (30)	2002	China	ROS	S	55.0(38.0-75.0)	61/25	48/20	1500	NA	conventional histopathological negative and IHC positive	Ber-EP4	25.0(1.0-67.0)	6, 7.0
Tanabe,T., (17)	2003	Japan	POS	S	63.0(40.0-78.0)	12/34	NA	3494	44(4-153) *	pN0 and IHC positive	AE1/AE3	54.0(7.0-136.0)	0
Shiozaki,H., (21)	2007	Japan	POS	M	NA	25/139	NA	NA	NA	pN0 and IHC positive	AE1/AE3	20.0	0
Chao,Y.K., (18)	2009	China	POS	S	57.2(39.0-68.0)	6/46	6/46	510	10(5–32)	pN0 and IHC positive	AE1/AE3	62.3(10.4-137.0)	0
Koenig,A.M., (6)	2009	Germany	ROS	S	60.0(39.0-83.0)	25/48	21/37	2174	25(6–74) *	conventional histopathological negative and IHC positive	AE1/AE3	38.5 (3.0-101.0)	4, 5.5
Zingg,U., (19)	2009	Switzerland	POS	S	61.0(36.0-85.0)	24/62	71/15	1204	NA	pN0 and IHC positive	AE1/AE3	47.4 (14.0-159.0)	3, 3.5
Thompson,S.K., (31)	2010	Australia	POS	S	61.3 ± 9.3	31/88	27/66	661	5*	pN0 and IHC positive	AE1/AE3	68.5	0
Prenzel,K.L., (32)	2012	Germany	POS	S	NA	7/41	NA	1344	28(15-52)	pN0 and IHC positive	AE1/AE3	99.6(58.8-160.8)	0
Chen,S.B., (5)	2020	China	POS	S	58.0(36.0-78.0)	88/428	65/301	11578	20 (10-69) *	pN0 and IHC positive	AE1/AE3	69.9 (1.0-174.0)	14, 2.7
Hiraki,Y. (7)	2021	Japan	RCT	M	67.0(46.0-78.0)	24/77	19/68	NA	67(16-139) *	conventional histopathological negative and IHC positive	AE1/AE3	60.0	NA

S/M ,single-center/multicenter; RCT, Randomized study; ROS, Retrospective observational study; POS, Prospective observational study; P/N, lymph node micrometastasis positive/lymph node micrometastasis negative; LNs, lymph nodes; IHC, Immunohistochemical; pN0, Node-negative; * median; pN0 and IHC positive: lymph node micrometastasis was defined as the presence in lymph nodes of tumor cells that were immunohistochemically positive for IHC staining. NA, Not available.

### 3.2 Quality assessment

Observational studies had low-to-moderate bias using the QUIPS tool (14) (**Supplementary Appendices 3**). Furthermore, confounding factors were not assessed in three studies (18, 27, 29); the drop-out time was not assessed in two studies (7, 26).

### 3.3 LNM and prognosis of esophageal cancer

**Figure 2:**
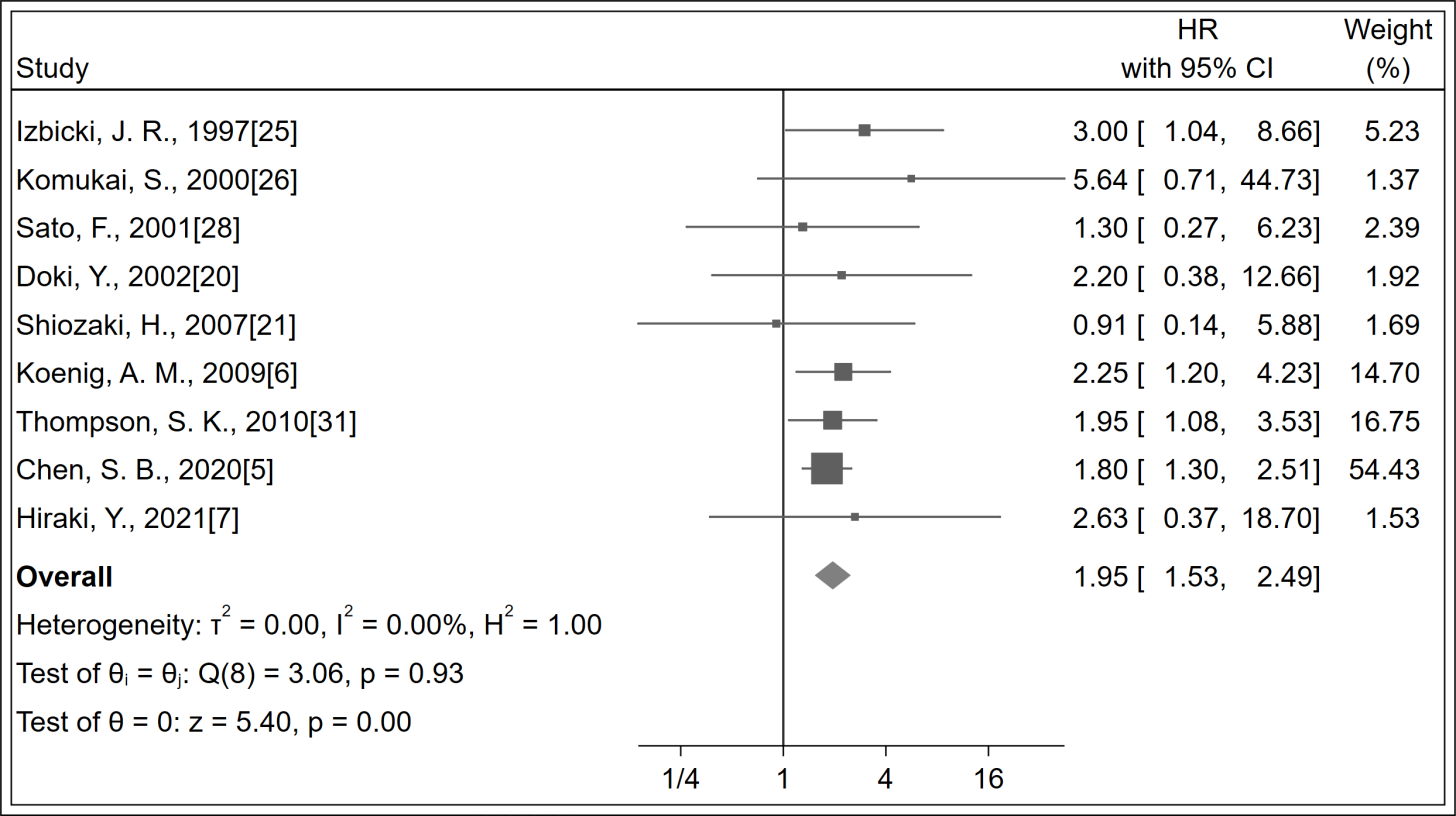
Forest plot of OS.

A total of 1,169 patients were identified in nine studies (5–7, 20, 21, 25, 26, 28, 31). The OS was significantly increased with LNM negativity compared with LNM positivity in esophageal cancer patients (HR 1.95; 95% CI, 1.53–2.49; *P <* 0.001; *I^2^* = 0.0%, *P* = 0.930; **Figure 2**; certainty of evidence, low). The results of subgroup analysis showed that there was a trend for OS in Asians (HR 1.82, 95% CI, 1.34–2.47; *I* ^2^ = 0.0%, *P* = 0.840) to be lower than in non-Asians (HR 2.20, 95% CI, 1.47–3.28; *I* ^2^ = 0.0%, *P* = 0.780), although no significant difference was observed between these subgroups (*P*=0.47) (**Table 2**) (**Supplementary Appendices 4**). Similarly, no significant differences were observed in outcomes between subgroups of study design, antibody types, follow-up duration, pN status, single-center/multicenter, univariate/multivariate analysis and tumor type (**Table 2**) (**Supplementary Appendices 5-11**).

**Table 2:** Subgroup analysis of clinical outcomes.

Subgroup	No. studies	No. patients	Effect size		Heterogeneity		Test of group differences
			HR (95%CI)		I^2^(%)	*P* value	*P* value
Region							
Asia	6	909	1.82(1.34,2.47)		0	0.84	0.47
Non-Asia	3	260	2.20(1.47,3.28)		0	0.78	
Study design							
RCT	1	101	2.63(0.37,18.70)		0	–	0.91
Prospective	5	164	1.91(1.45,2.50)		0	0.65
Retrospective	3	904	2.10(1.20,3.65)		0	0.82
Antibody types							
AE1/AE3	8	1101	1.91(1.49,2.44)		0	0.93	0.41
Ber-EP4	1	68	3.00(1.04,8.66)		0	–
Follow-up duration							
Less than 5 years	2	232	2.14(0.74,6.14)		15.78	0.28	0.85
More than 5 years	7	937	1.93(1.50,2.49)		0	0.94
Single-center/multicenter							
Single-center	6	863	1.96(1.53,2.52)		0	0.81	0.83
Multicenter	3	306	1.74(0.60,5.06)		0	0.70
**Tumor types**							
SCC+EAC	3	260	2.20(1.47,3.28)		0	0.78	0.47
SCC	6	909	1.82(1.34,2.47)		0	0.84
Univariate/multivariate analysis							
Multivariate analysis	5	790	1.91(1.46,2.51)		0	0.72	0.76
Univariate analysis	4	379	2.10(1.22,3.63)		0	0.83
pN status							
pN+	3	242	2.44(1.45,4.12)		0	0.90	0.34
pN0	6	927	1.83(1.40,2.41)		0	0.86
Relapse-free survival							
Region							
Asia	5	803	3.18(1.43,7.05)		54.53	0.07	0.61
Non-Asia	2	141	4.25(1.93,9.37)		0	0.68
Study design							
RCT	1	101	4.75(1.13,19.90)	–		–	0.85
Prospective	4	707	2.99(1.50,5.98)	52.34		0.10
Retrospective	2	136	3.50(0.53,23.06)	59.03		0.12
Antibody types							
AE1/AE3	5	790	2.73(1.41,5.29)	37.66		0.17	0.25
Ber-EP4	2	154	4.94(2.28,10.73)	0		0.38
Follow-up duration							
Less than 5 years	2	154	4.94(2.28,10.73)	0		0.38	0.25
More than 5 years	5	790	2.73(1.41,5.29)	37.66		0.17
Single-center/multicenter							
Single-center	6	843	3.29(1.71,6.33)	54.03		0.05	0.65
Multicenter	1	101	4.75(1.13,19.90)	–		–
Tumor types							
SCC+EAC	3	240	5.03(2.58,9.82)	0		0.68	0.10
SCC	4	704	2.28(1.19,4.37)	26.96		0.25
Univariate/multivariate analysis							
Multivariate analysis	6	843	3.29(1.71,6.33)	54.03		0.05	0.65
Univariate analysis	1	101	4.75(1.13,19.90)	0		0.06
pN status							
pN+	3	255	4.90(2.48,9.69)	0		0.68	0.19
pN0	4	689	2.49(1.19,5.22)	38.41		0.18

HR, hazard Ratio; RCT, randomized controlled trial; SCC, Squamous cell carcinoma; EAC, Esophageal adenocarcinoma.

**Figure 3:**
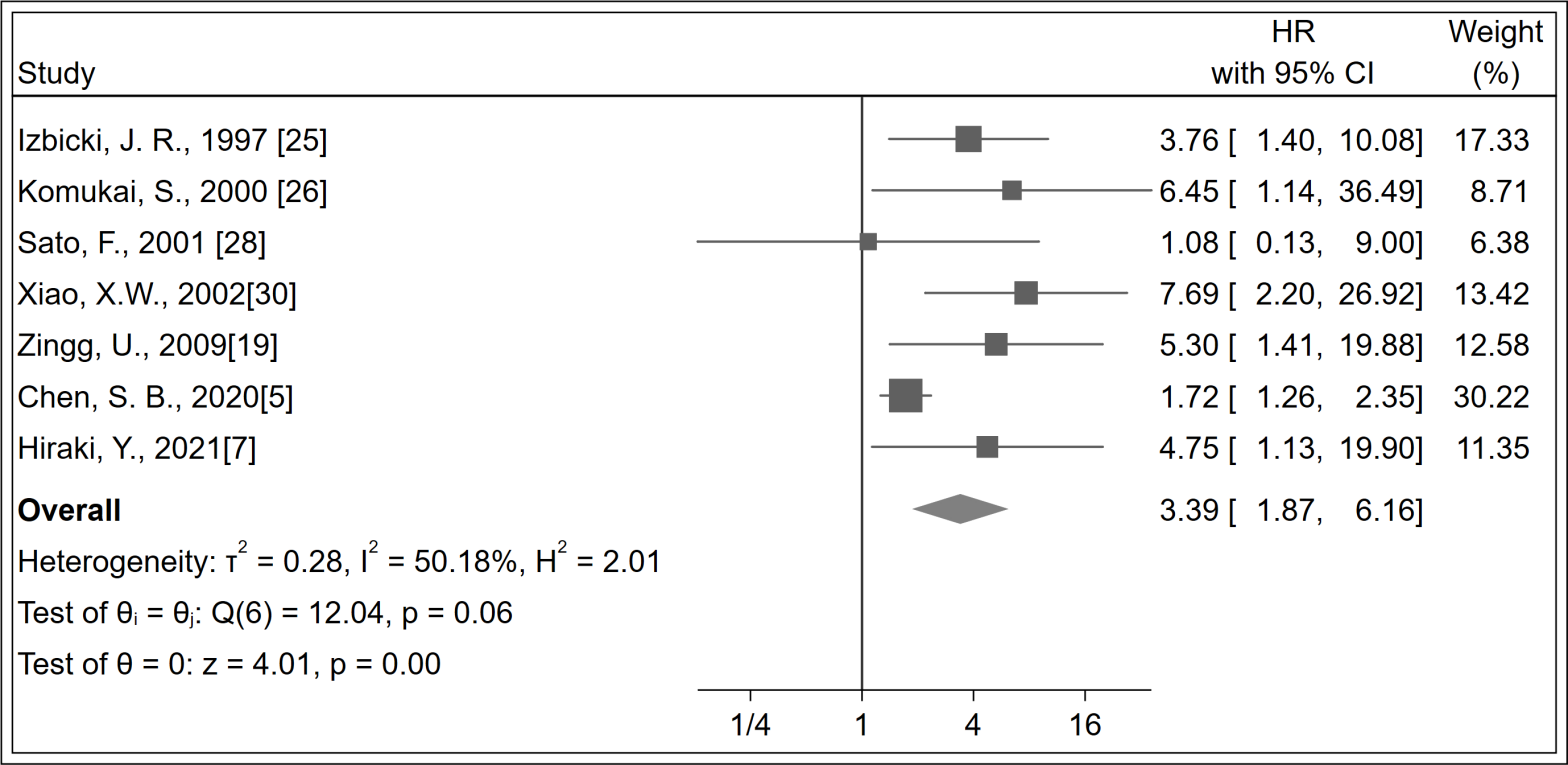
Forest plot of RFS.

A total of 944 patients were identified in seven studies (5, 7, 19, 25, 26, 28, 30). The RFS was significantly increased with LNM negativity compared with LNM positivity in esophageal cancer patients (HR 3.39; 95% CI, 1.87–6.16; *P <* 0.001; *I^2^* = 50.18%, *P* = 0.060; **Figure 3**; certainty of evidence, moderate). The results of subgroup analysis showed that there was a trend for RFS in Asians (HR 3.18, 95% CI, 1.43–7.05; *I* ^2^ = 54.53%, *P* = 0.070) to be lower than in non-Asians (HR 4.25, 95% CI, 1.93–9.37; *I* ^2^ = 0.0%, *P* = 0.680), although no significant difference was seen between these subgroups (*P* = 0.610) (**Table 2** **) (** **Supplementary Appendices 12**). Further, no significant differences were observed between subgroups of study design, region, antibody types, follow-up duration, pN status, single-center/multicenter, univariate/multivariate analysis, and tumor types (**Table 2**) (**Supplementary Appendices 13-19**).

**Figure 4:**
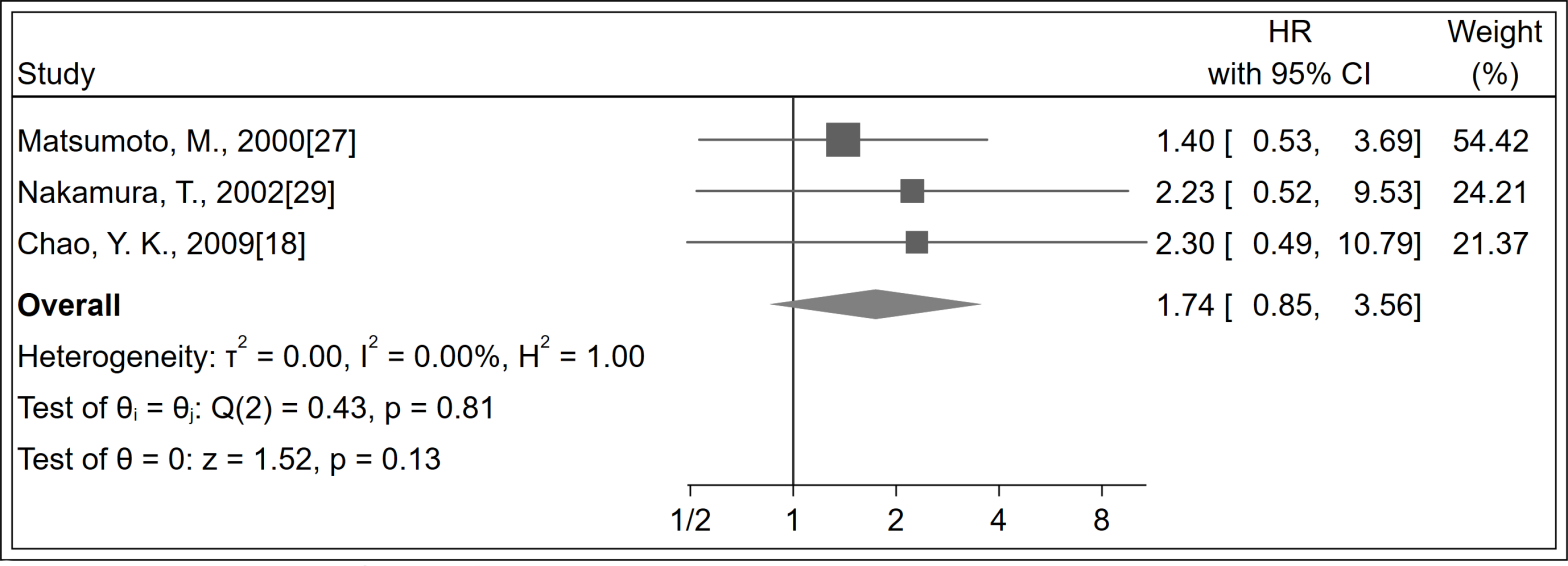
Forest plot of recurrence.

A total of 184 patients were identified in three studies (18, 27, 29). No significant difference was observed in recurrence between LNM positivity and LNM negativity groups (HR 1.74; 95% CI, 0.85–3.56; *P* = 0.130; *I^2^* = 0%, *P* = 0.810; **Figure 4**; certainty of evidence, low). Further, no significant differences were observed between subgroups of follow-up duration (**Supplementary Appendices 20**).

### 3.4 Sensitivity analysis and publication bias

Sensitivity analysis with the exclusion of one trial at a time revealed a stable trend (**Supplementary Appendices 21-23**). In addition, after excluding three articles (6, 20, 21) for which the HR was transformed by a survival curve, it was observed that OS was significantly increased with LNM negativity compared with LNM positivity (HR 1.93; 95% CI, 1.47–2.52; *P* < 0.001; *I^2^* = 0.0%, *P* = 0.820), and the trend was still stable.

**Figure 5:**
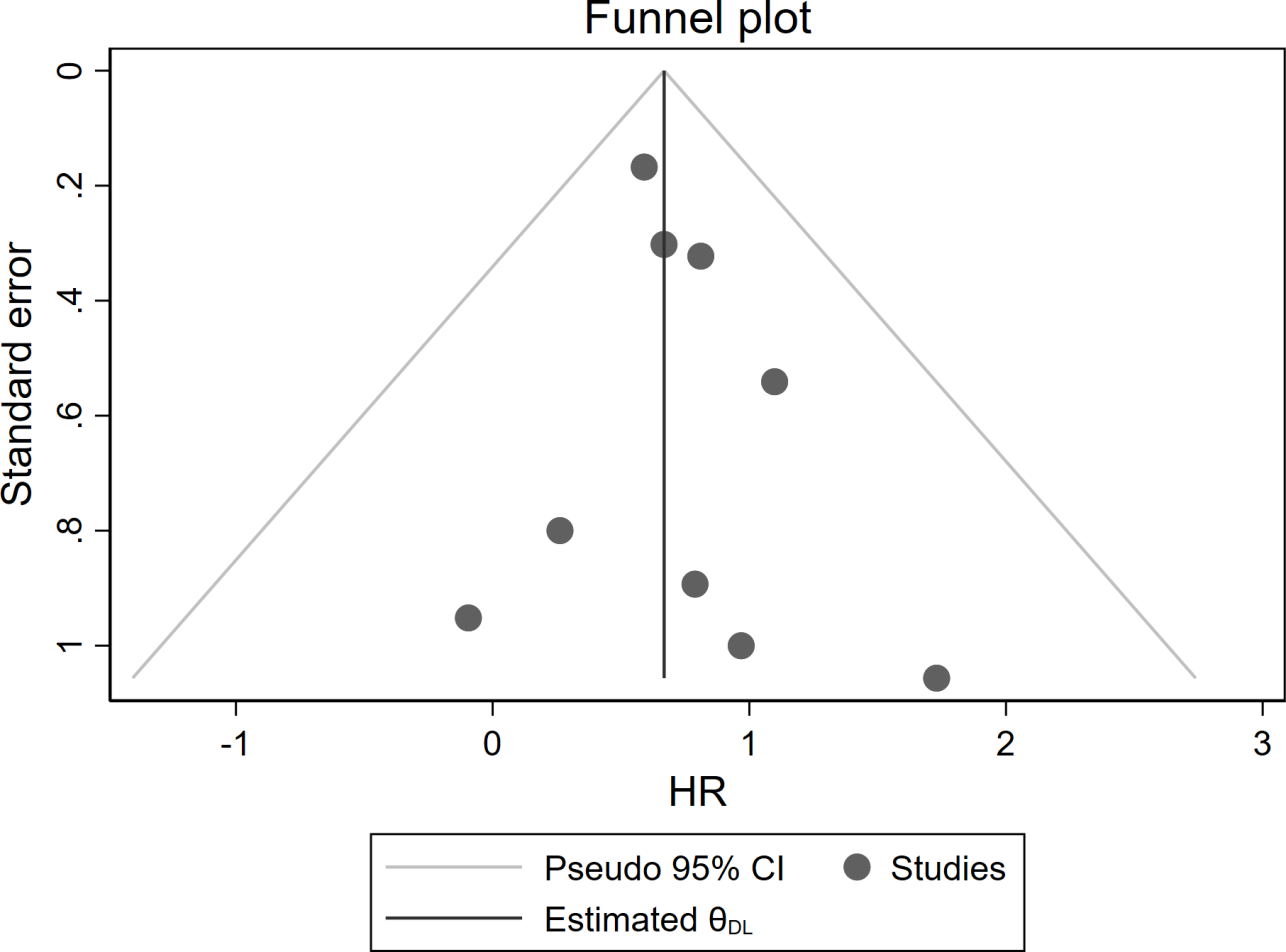
Funnel plot to assess publication bias of OS.

Moreover, no significant asymmetry was observed by visual inspection of the funnel plot of studies reporting OS (**Figure 5**). The Egger’s test did not show significant publication bias (*P* = 0.445).

### 3.5 Evidence quality

The original studies were observational studies that provided low-quality evidence. The OS and recurrence data showed low certainty, indicating that our confidence in the effect estimate is limited: the true effect may be substantially different from the estimate of the effect (33, 34). The outcome of RFS was upgraded because of the large effect size; therefore, it shows moderate certainty, indicating that we are moderately confident in the effect estimate: the true effect is likely to be close to the estimate of the effect, but there is a possibility that it is substantially different (33, 34). Further details are provided in **Table 3**.

**Table 3:** Summary of the results of GRADE.

Certainty assessment							№ of patients		Effect		Certainty
№ of studies	Study design	Risk of bias	Inconsistency	Indirectness	Imprecision	Other considerations	LNM positive	LNM negative	Relative(95% CI)	Absolute(95% CI)	
Overall survival (follow-up: range 1 months to 174 months)											
9	observational studies	not serious	not serious	not serious	not serious	none	279/1169 (23.9%)	890/1169 (76.1%)	HR 1.95 (1.53 to 2.49)	177 more per 1,000 (from 127 more to 210 more)	⨁⨁◯◯ Low
Relapse-free survival (follow-up: range 1 months to 174 months)											
7	observational studies	not serious	not serious	not serious	not serious	strong association	272/944 (28.8%)	672/944 (71.2%)	HR 3.39 (1.87 to 6.16)	273 more per 1,000 (from 191 more to 288 more)	⨁⨁⨁◯ Moderate
Recurrence (follow-up: range 10.4 months to 137 months)											
3	observational studies	not serious	not serious	not serious	not serious	none	53/194 (27.3%)	141/194 (72.7%)	OR 1.74 (0.85 to 3.56)	96 more per 1,000 (from 33 fewer to 178 more)	⨁⨁◯◯ Low

LNM positive: lymph node micrometastasis positive; LNM negative: lymph node micrometastasis negative; The risk in the intervention group (and its 95% confidence interval) is based on the assumed risk in the comparison group and the relative effect of the intervention (and its 95% CI). CI, confidence interval; HR, hazard Ratio; OR, odds ratio CI, confidence interval; HR, hazard Ratio; OR, odds ratio.

## 4 Discussion

In this meta-analysis, moderate-quality evidence showed that RFS was significantly increased with LNM negativity compared with LNM positivity in esophageal cancer patients. The OS was significantly increased with LNM negativity compared with LNM positivity in esophageal cancer patients, although the quality of evidence was low. However, no significant difference was observed in recurrence. Regretfully, several subgroups were not found to be a source of heterogeneity.

According to the UICC Tumor, Node, Metastasis-classification, eighth editions guidelines on lymph node involvement in esophageal cancer, the presence of tumor cells exceeding 0.2 mm in greatest extent is categorized as metastasis (9). LNM is a tumor lesion in a lymph node between 0.2 and 2 mm in diameter and/or a microscopic collection of more than 200 tumor cells in a lymph node (35–37).

Although most studies (19, 31, 32) have found that LNM positivity indicates a worse prognosis than LNM negativity, studies (7, 28) have found no statistically significant difference between prognosis between the two. Further, these studies (27, 29, 38) have found that recurrence with LNM positivity has a different clinical significance in esophageal cancer. Most of the included observational trials had small statistical power in this meta-analysis. Meta-analysis is an ideal statistical tool that increases the statistical power and the precision of comparisons and offers more powerful evidence for clinical decision-making. Thus, this meta-analysis was conducted to assess the clinical significance of LNM in esophageal cancer. Our meta-analysis showed that LNM is a strong prognostic factor in esophageal cancer.

We also conducted subgroup analyses in this meta-analysis including subgroups of study design, region, antibody type, follow-up duration, pN status, single-center/multicenter study, univariate/ultivariate analysis, and tumor type to explore heterogeneity. However, we could not identify the source of heterogeneity. Among the subgroups, it found that OS and RFS may be a worse trend for non-Asians compared with Asians. The incidence of esophageal cancer has increased over the past years in the Western world and is predicted to increase further given a rise in alcohol consumption and lack of physical exercise (39). Compared with Eastern patients, Western patients have a larger BMI, making it relatively difficult to achieve the minimal number of harvested lymph nodes. This is associated with a worse prognosis for esophageal cancer in Western patients (40, 41).

The results of this study are of clinical significance. Here we provide a more objective appraisal of the evidence than traditional narrative reviews and a more precise estimate of the prognostic value of LNM than that currently available. The data presented here may help plan future clinical trials and may help determine whether LNM can be used as a prognostic factor in esophageal cancer. For example, LNM was included in the current AJCC staging system for breast cancer (35); however, LNM in esophageal cancer is not designated as a staging parameter. The data from observational studies suggest that LNM has a clinically significant detrimental effect on OS and RFS in esophageal cancer. It has been shown that control of LNM by neoadjuvant chemotherapy (NAC) in esophageal squamous cell carcinoma (ESCC) is significantly associated with improved RFS at pN0 stage (7). Patients with ESCC who underwent surgery after receiving NAC with Adriamycin + cisplatin + 5-fluorouracil (ACF) or docetaxel + cisplatin + 5-fluorouracil (DCF) have shown a better RFS in the DCF group, and DCF controlled LNM better than ACF (7, 42). In addition to controlling distant metastasis recurrence, LNM control is an important requirement in NAC regimens, and DCF is effective for LNM control (42, 43). We ventured to speculate whether LNM can be included in the staging of N in esophageal cancer. At the same time, LNM was a poor prognostic factor in esophageal cancer, which provided a reference for the treatment and prognostic value of LNM in other gastrointestinal cancers.

There were some limitations to this meta-analysis. First, because the definition of LNM itself has not been standardized, the original research reported some differences in the definition of LNM, which may be the source of heterogeneity. However, further exploration could not be carried out because of the limitation of the original data. Second, an estimated moderate degree of heterogeneity was found for RFS among studies. However, secondary analyses including subgroup analysis and sensitivity analysis were performed to partly explore this limitation. Third, the results of this study needed to be interpreted with caution because a majority of studies were Asian, although there were no restrictions on the study population in this study. Lastly, the evidence is low-to-moderate quality because most studies included in the analysis were observational in nature. Therefore, future relevant high-quality studies with a large sample size are needed to confirm the prognostic value of LNM.

## 5 Conclusion

LNM positivity has a worse prognosis in esophageal cancer, although the evidence for this is low to moderate. Future relevant high-quality studies are needed to further validate our results and provide a reference for guidelines.

## Data availability statement

The raw data supporting the conclusions of this article will be made available by the authors, without undue reservation.

## Author contributions

JY and QL: literature search, screening, data extraction, data analysis and results visualization. The manuscript was written with the contributions of all authors. MZ: fund acquirement. All authors have approved the final version of the manuscript.
