# Supplementary material for: Prognostic value of lymph node micrometastasis in esophageal cancer: A systematic review and meta-analysis
Source: Front Oncol. 2023 Jan 4;12:1025855. doi: 10.3389/fonc.2022.1025855 (PMC9845692; doi:10.3389/fonc.2022.1025855)

Catalogs

Online supplements 2

Prognostic value of lymph node micrometastasis in esophageal cancer: A systematic review and meta-analysis 2

Supplementary Appendices 1 Literature search strategy of Pubmed 2

Supplementary Appendices 2 Basic features of esophageal cancer 3

Supplementary Appendices 3 Quality in Prognosis Studies tool 4

Supplementary Appendices 4 Forest plot of region subgroup of OS 5

Supplementary Appendices 5 Forest plot of study design subgroup of OS 6

Supplementary Appendices 6 Forest plot of antibody type subgroup of OS 7

Supplementary Appendices 7 Forest plot of follow-up duration subgroup of OS 8

Supplementary Appendices 8 Forest plot of single-center/multicenter subgroup of OS .. 9

Supplementary Appendices 9 Forest plot of tumor type subgroup of OS 10

Supplementary Appendices 10 Forest plot of univariate/multivariate analysis subgroup of OS 11

Supplementary Appendices 11 Forest plot of pN status subgroup of OS 12

Supplementary Appendices 12 Forest plot of region subgroup of RFS 13

Supplementary Appendices 13 Forest plot of study design subgroup of RFS 14

Supplementary Appendices 14 Forest plot of antibody type subgroup of RFS............... 15

Supplementary Appendices 15 Forest plot of follow-up duration subgroup of RFS 16

Supplementary Appendices 16 Forest plot of single-center/multicenter subgroup of RFS 17

Supplementary Appendices 17 Forest plot of tumor type subgroup of RFS 18

Supplementary Appendices 18 Forest plot of univariate/multivariate analysis subgroup of RFS 19

Supplementary Appendices 19 Forest plot of pN status subgroup of RFS 20

Supplementary Appendices 20 Forest plot of follow-up duration subgroup of recurrence 20

Supplementary Appendices 21 Sensitivity analysis of OS 21

Supplementary Appendices 22 Sensitivity analysis of RFS 21

Supplementary Appendices 23 Sensitivity analysis of recurrence 22

Online supplements

Prognostic value of lymph node micrometastasis in esophageal cancer: A systematic review and meta-analysis

Supplementary Appendices 1 Literature search strategy of Pubmed

(("Neoplasm Micrometastasis"[Mesh]) OR (((((((Neoplasm Micrometastasis[Title/Abstract]) OR (Neoplasm Micrometastases[Title/Abstract])) OR (Neoplasm Micrometastase[Title/Abstract])) OR (Micrometastasis[Title/Abstract])) OR (Micrometastases[Title/Abstract])) OR (micro-metastases[Title/Abstract])) OR (micro-metastasis[Title/Abstract]))) AND ((("Esophageal Neoplasms"[Mesh]) OR (((((((((((((((Esophageal Neoplasms[Title/Abstract]) OR (Esophageal Neoplasm[Title/Abstract])) OR (Esophagus Neoplasm[Title/Abstract])) OR (Esophagus Neoplasms[Title/Abstract])) OR (Cancer of Esophagus[Title/Abstract])) OR (Cancer of the Esophagus[Title/Abstract])) OR (Esophagus Cancer[Title/Abstract])) OR (Esophagus Cancers[Title/Abstract])) OR (Esophageal Cancer[Title/Abstract])) OR (Esophageal Cancers[Title/Abstract])) OR (esophageal tumor[Title/Abstract])) OR (esophagus tumour[Title/Abstract])) OR (oesophageal neoplasms[Title/Abstract])) OR (oesophageal tumor[Title/Abstract])) OR (oesophagus tumor[Title/Abstract])) OR (oesophagus tumour[Title/Abstract])))

Supplementary Appendices 2 Basic features of esophageal cancer

First author	Tumor invasion P/N	Site of tumor P/N	Grade of differentiation P/N	Type of tumor	Adjustment factors
Izbicki,J.R. [25]	Tis 1/1; T1 4/5; T2 9/10; T3 25/10; T4 3/0	NA	NA	SCC+ EAC	Lymph-node micrometastasis, histopathological lymph-node stage, histologic tumor grade and lymphovascular invasion, primary-tumor stage and tumor type
Komukai,S., [26]	T1 3/12; T2 3/3; T3 8/8	Upper 0/3; Middle 9/15; Lower 2/3	Well 3/5; Mod 9/15; Poor 2/3	SCC	Sex, age, primary site, grade of differentiation, T category, lymphatic invasion, vascular
Matsumoto, M.,[27]	T1 1/22; T2 9/2; T3 23/2	Upper 4/7; Middle 17/9; Lower 12/10	Well 13/6; Mod 15/12; Poor 5/6	SCC	NA
Sato,F.,[28]	T1 9/17; T2 2/6; T3-4 9/7	Cervical 0/1; Upper 0/5; Middle 15/20; Lower 5/4	Well 6/7; Mod 9/16; Poor 5/7	SCC	Sex, age, primary site, grade of differentiation, T category
Doki,Y., [20]	T3 7/19; T4 4/11	Upper 1/4; Middle 5/15; Lower 5/11	Well 4/9; Mod 5/15; Poor 2/6	SCC	NA
Nakamura,T .,[29]	T1 6/24; T2 4/4; T3 4/11	Upper 1/3; Middle 11/30; Lower 2/6	Well 6/11; Mod 7/20; Poor 1/8	SCC	NA
Xiao,X.W. [30]	T1 12/6; T2 18/9; T3 30/10; T4 1/0	Supracarinal 33/17; Infracarinal 28/8	Well 1/0; Mod 44/21; Poor 16/4	SCC+ EAC	Ber-EP4 (negative v positive), lymph-node stage, T category, grade of differentiation, tumor type, skip metastasis, skip micrometastasis, combined skip metastasis
Tanabe,T., [17]	Mucosal4/15; Submucosal 8/19	NA	Well 10/33; Poor 2/1	SCC	Multicentric lesion, depth of invasion, growth pattern, grade of differentiation, cellular atypia, lymphatic invasion, venous invasion
Shiozaki,H., [21]	T2 11/49; T3 12/75	Upper 0/18; Middle 18/92; Lower 7/29	Well 10/59; Mod 10/60; Poor 5/20	SCC	NA
Chao,Y.K., [18]	NA	C+U 8; M+L 44	NA	SCC	NA
Koenig, A. M.,[6]	T1 7/24; T2 7/19; T3 11/30	NA	Well 8/16; Mod 11/40; Poor 6/17	SCC+ EAC	Age, sex, T stage, N stage, ratio of nodal microinvolvement
Zingg,U., [19]	T0 1/21; T1 1/21; T2 7/14; T3 7/14	NA	Well 0/6; Mod 18/43; Poor 6/13	SCC+ EAC	Pre-treatment; micrometastasis, UICC stage, tumor type
Thompson,S .K.,[31]	T0 2/17; Tis 0/6; T1 6/30; T2 7/8; T3 13/27	Middle 1/18; Lower 7/33; GOJ 23/37	Well +Mod 8/45; Poor 22/35	SCC+ EAC	Age, sex, histologic type, vascular invasion, and the presence of occult tumor deposits
Prenzel,K.L. ,[32]	T1a/T1b 20/49	NA	Mod 53; Poor 16	SCC+ EAC	NA
Chen,S.B., [5]	T1 5/75; T2 19/101; T3 53/216; T4 11/36	Upper 10/67; Middle 70/301; Lower 8/60	Well 24/181; Mod 54/211; Poor 10/36	SCC	Gender, age, histologic grade, T category
Hiraki,Y., [7]	T1 0/2; T2 6/14; T3 18/61	Upper 3/8; Middle 11/41; Lower 10/28	NA	SCC	Regimen ACF, Number of LNs dissected, T category, N category, M category, pathological response grade, micrometastasis

C cervical esophagus; U upper third thoracic; M middle third thoracic esophagus; L lower third thoracic esophagus; Mod Moderate; EAC esophageal adenocarcinoma; SCC squamous cell carcinoma;

Supplementary Appendices 3 Quality in Prognosis Studies tool

First author	Year	Study participation	Study attrition	Prognostic factor measurement	Outcome measurement	Confounding measurement and account	Analysis	Risk of bias
Izbicki,J.R.[25]	1997	Low	Low	Low	Low	Low	Low	Low
Komukai,S.,[26]	2000	Low	Moderate	Low	Low	Low	Low	Low
Matsumoto,M.,[27]	2000	Low	Low	Low	Low	Moderate	Low	Moderate
Sato,F.,[28]	2001	Low	Low	Low	Low	Low	Low	Low
Doki,Y.,[20]	2002	Low	Low	Low	Low	Low	Low	Low
Nakamura,T.,[29]	2002	Low	Low	Low	Low	Moderate	Low	Moderate
Xiao,X.W.[30]	2002	Low	Low	Low	Low	Low	Low	Low
Tanabe,T., [23]	2003	Low	Low	Low	Low	Low	Low	Low
Shiozaki,H.,[21]	2007	Low	Low	Low	Low	Low	Low	Low
Chao,Y.K.,[18]	2009	Low	Low	Low	Low	Moderate	Low	Moderate
Koenig,A.M.,[6]	2009	Low	Low	Low	Low	Low	Low	Low
Zingg,U.,[19]	2009	Low	Low	Low	Low	Low	Low	Low
Thompson,S.K.,[31]	2010	Low	Low	Low	Low	Low	Low	Low
Prenzel,K.L.,[32]	2012	Low	Low	Low	Low	Low	Low	Low
Chen,S.B.,[5]	2020	Low	Low	Low	Low	Low	Low	Low
Hiraki,Y.,[7]	2021	Low	Moderate	Low	Low	Low	Low	Low

Supplementary Appendices 4 Forest plot of region subgroup of OS

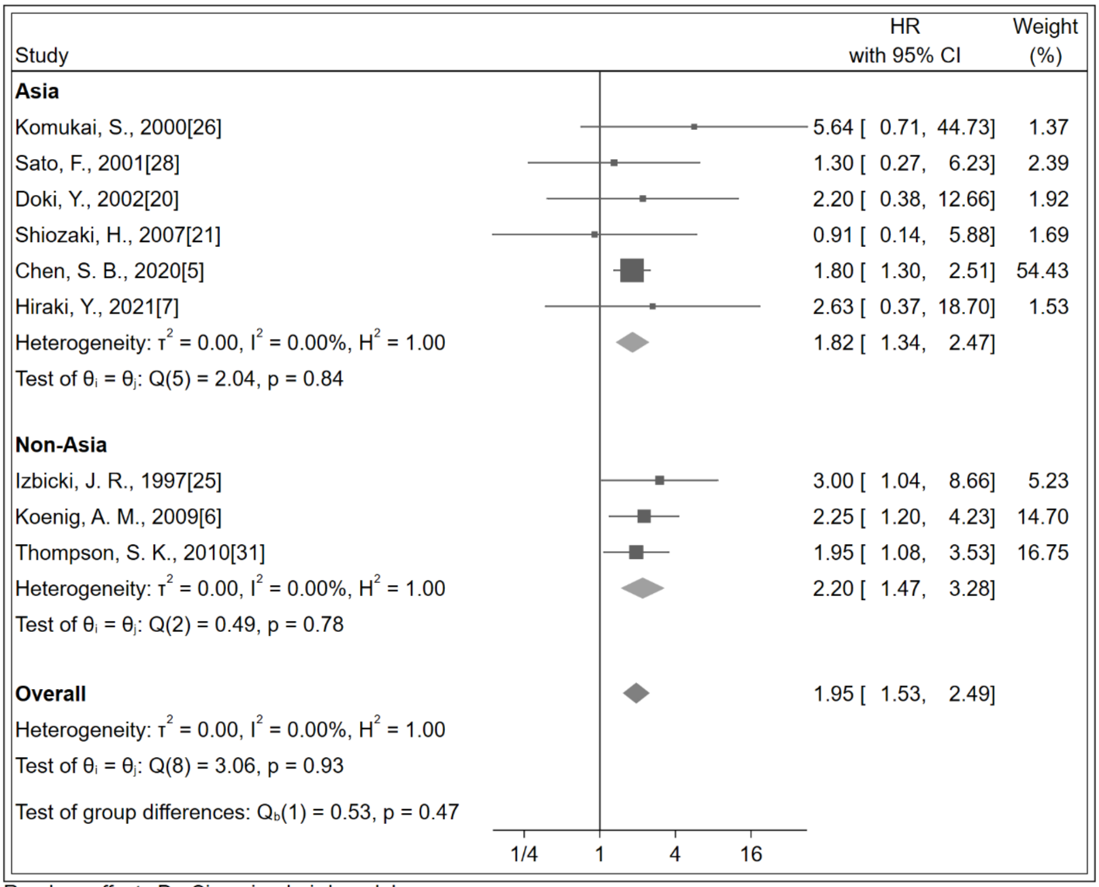

Random-effects DerSimonian-Laird model

Supplementary Appendices 5 Forest plot of study design subgroup of OS

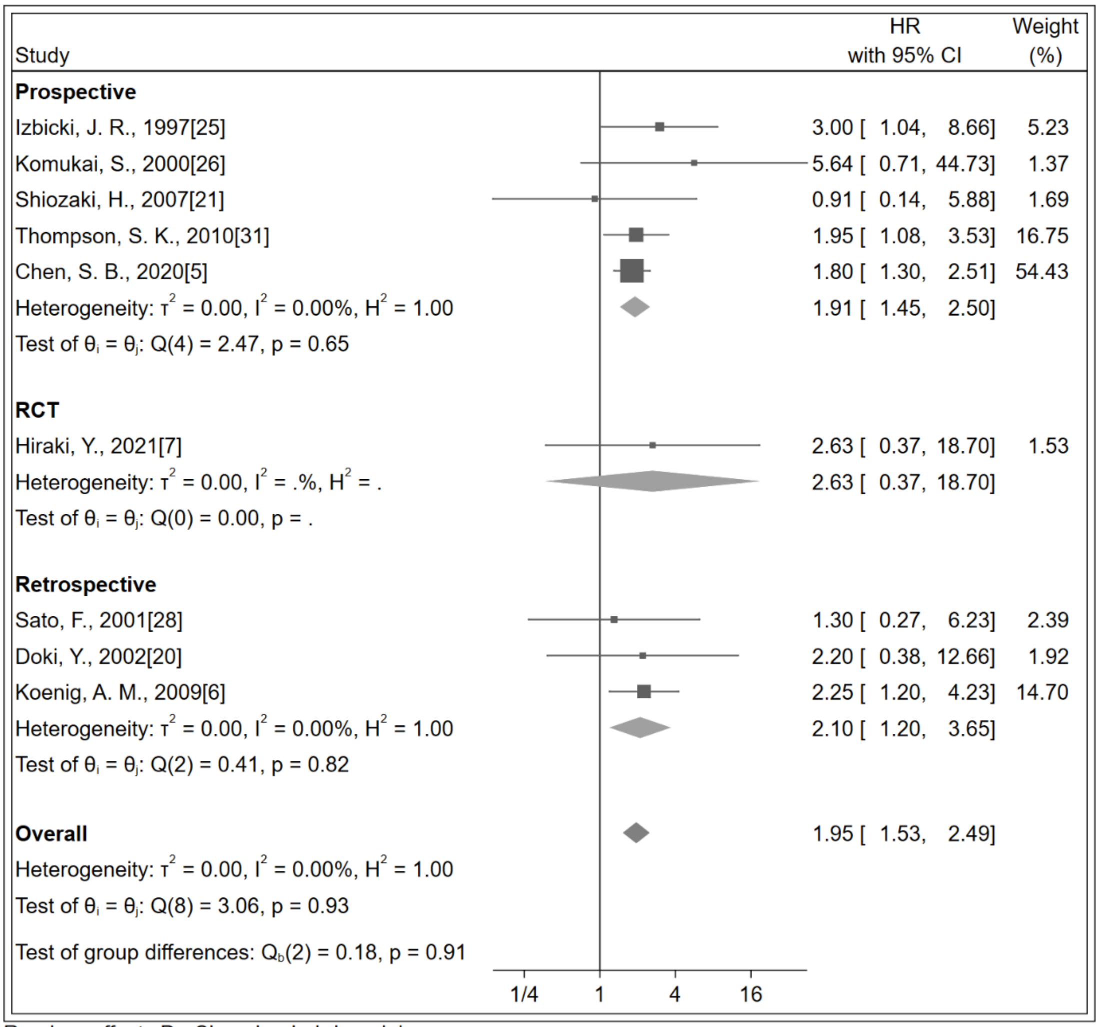

Random-effects DerSimonian-Laird model

Supplementary Appendices 6 Forest plot of antibody type subgroup of OS

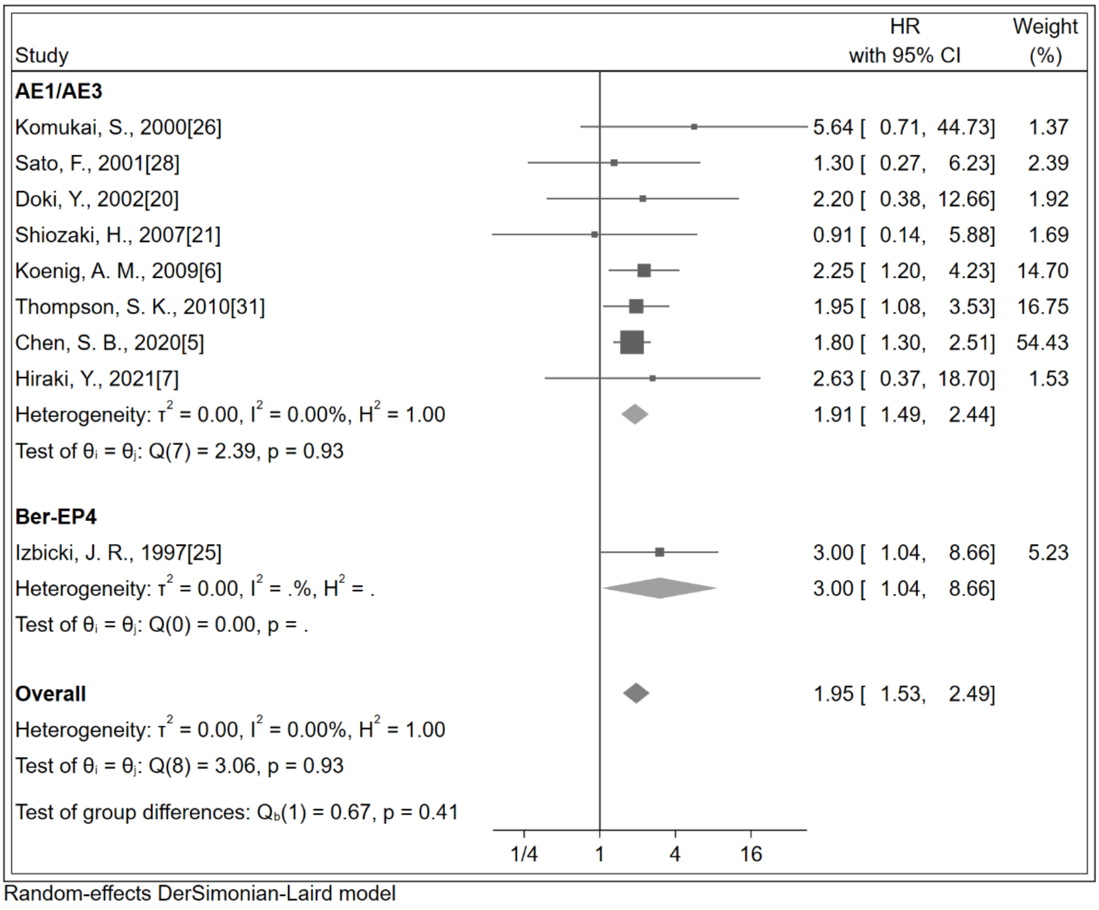

Supplementary Appendices 7 Forest plot of follow-up duration subgroup of OS

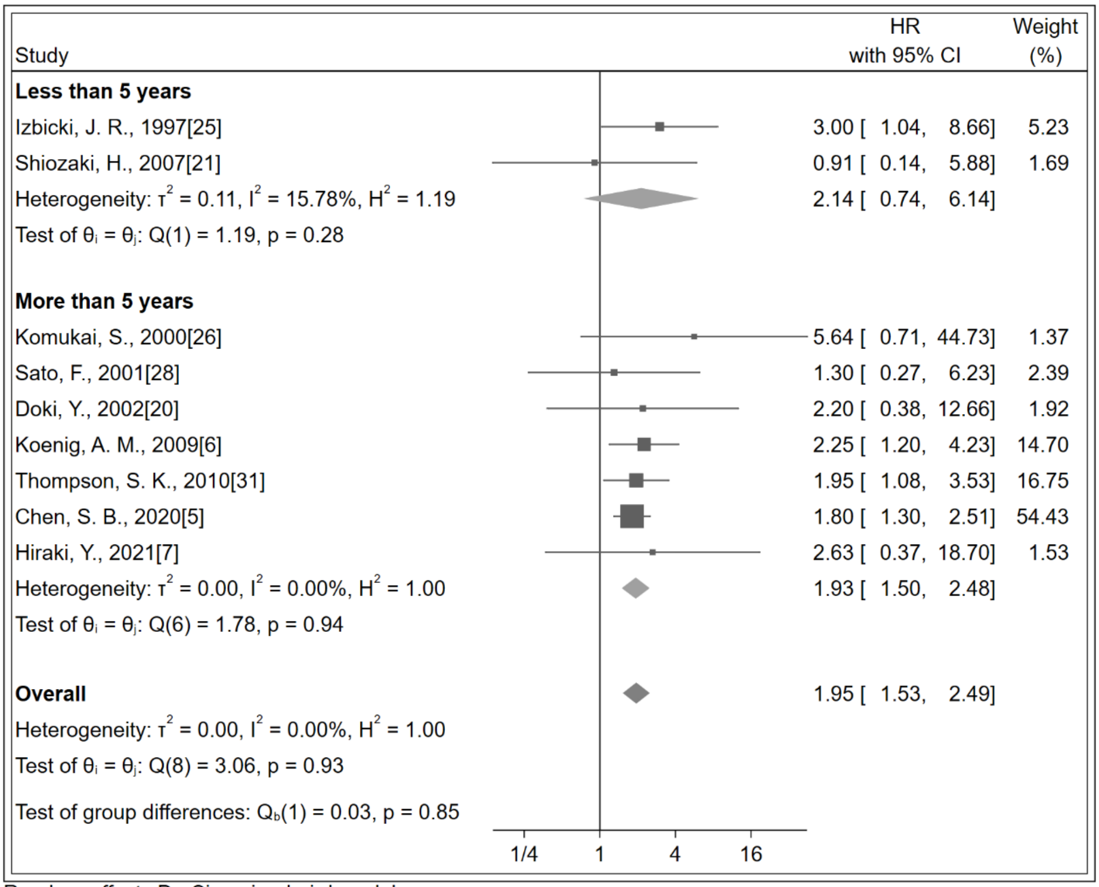

Random-effects DerSimonian-Laird model

Supplementary Appendices 8 Forest plot of single-center/multicenter subgroup of OS

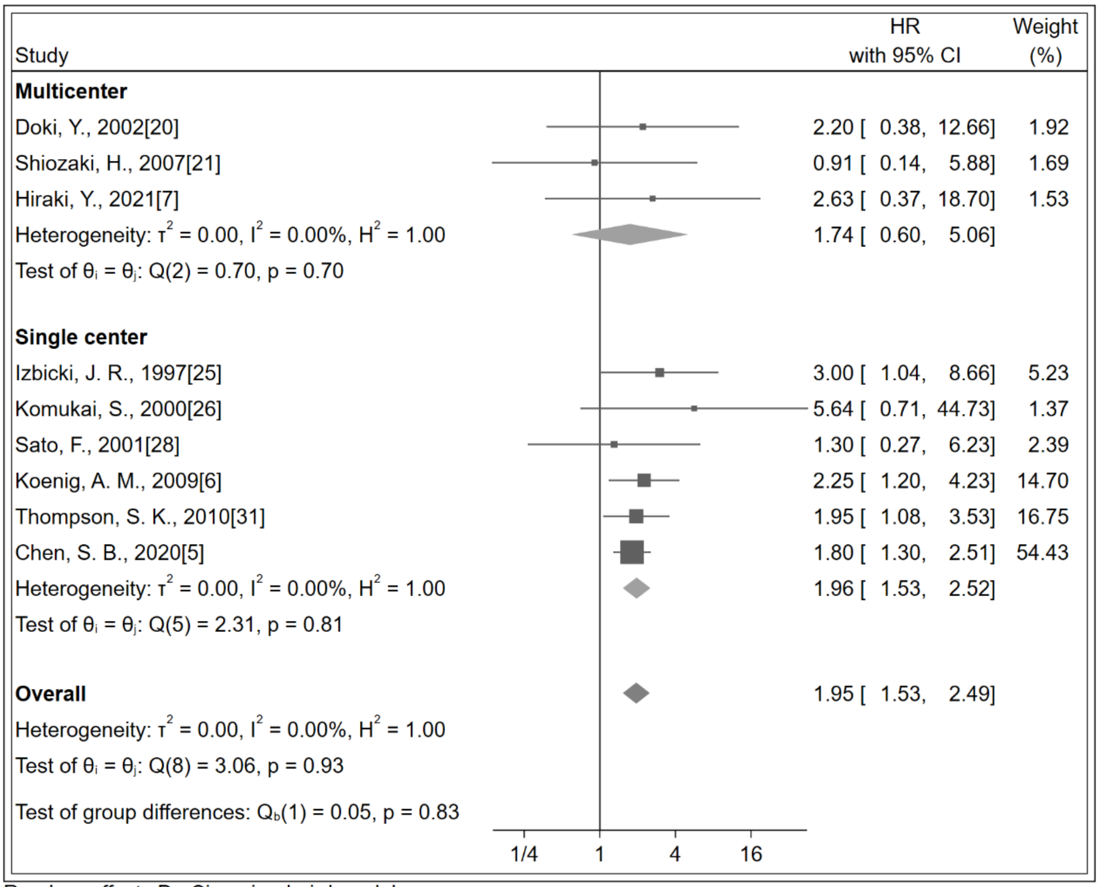

Random-effects DerSimonian-Laird model

Supplementary Appendices 9 Forest plot of tumor type subgroup of OS

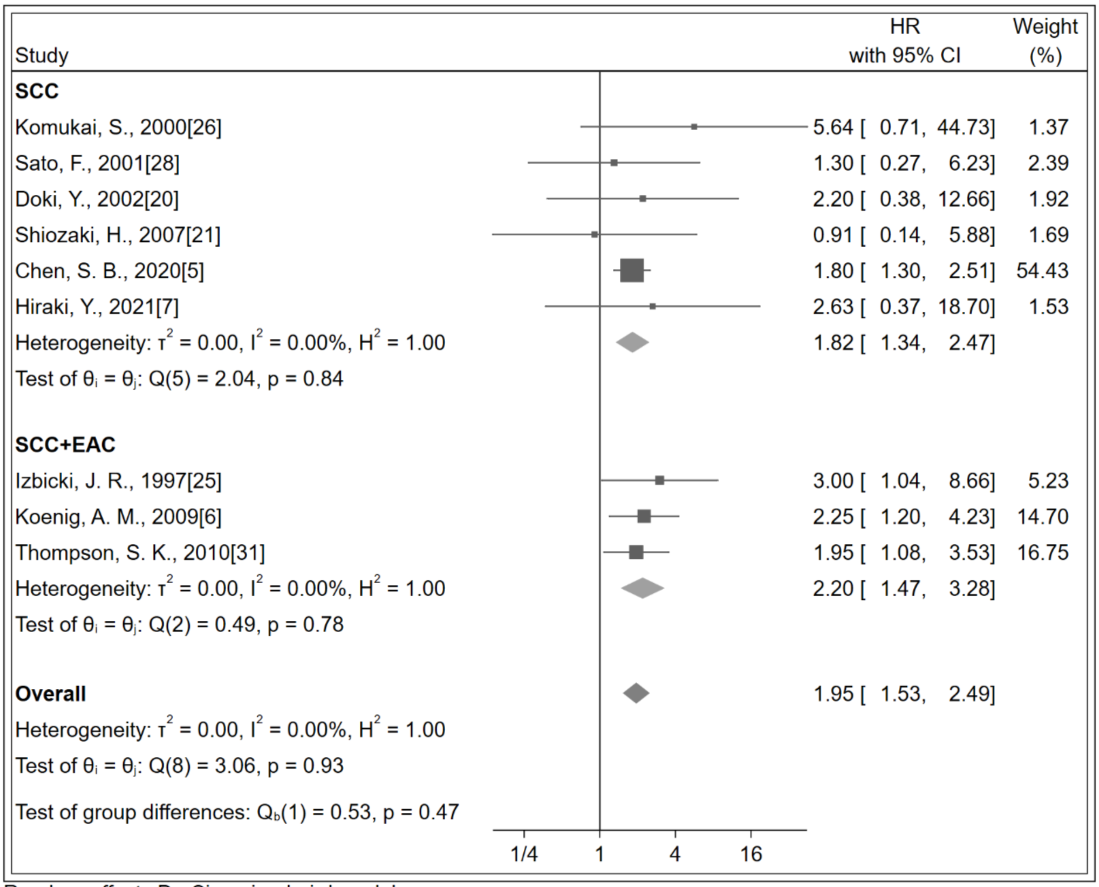

Random-effects DerSimonian-Laird model

Supplementary Appendices 10 Forest plot of univariate/multivariate analysis subgroup of OS

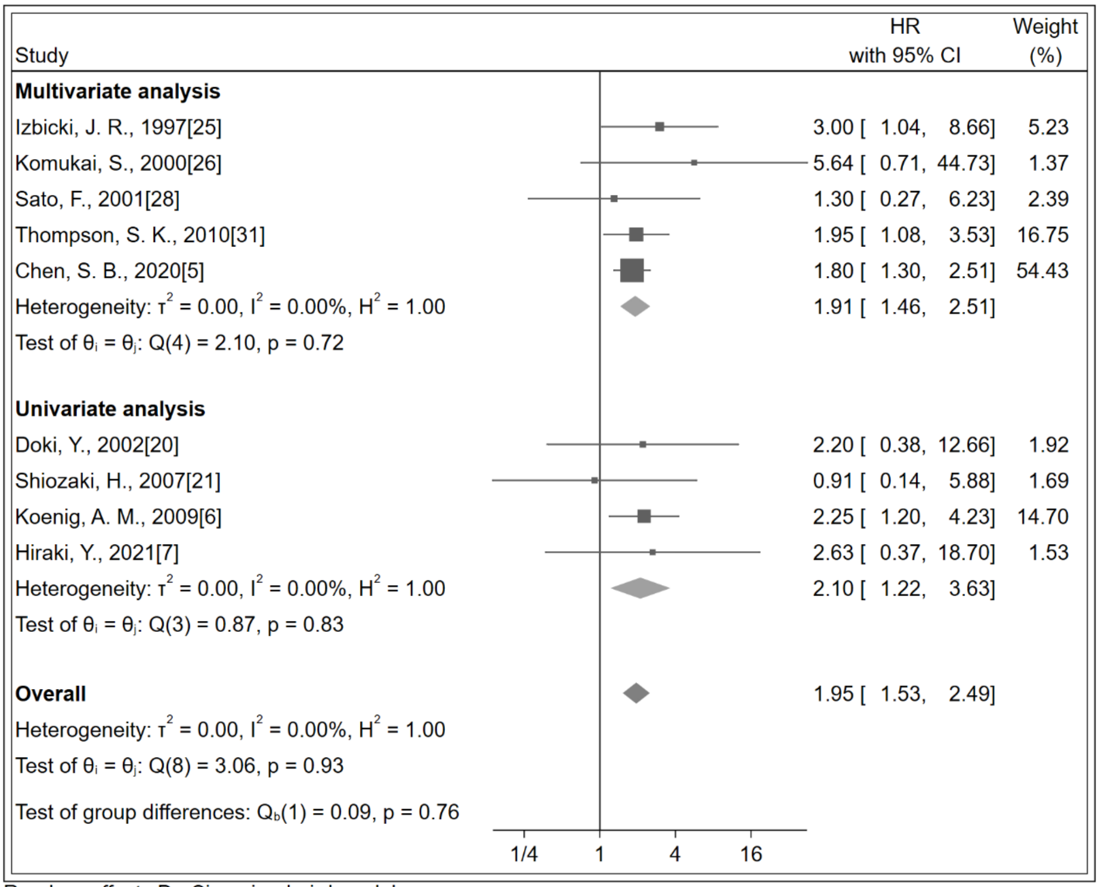

Random-effects DerSimonian-Laird model

Supplementary Appendices 11 Forest plot of pN status subgroup of OS

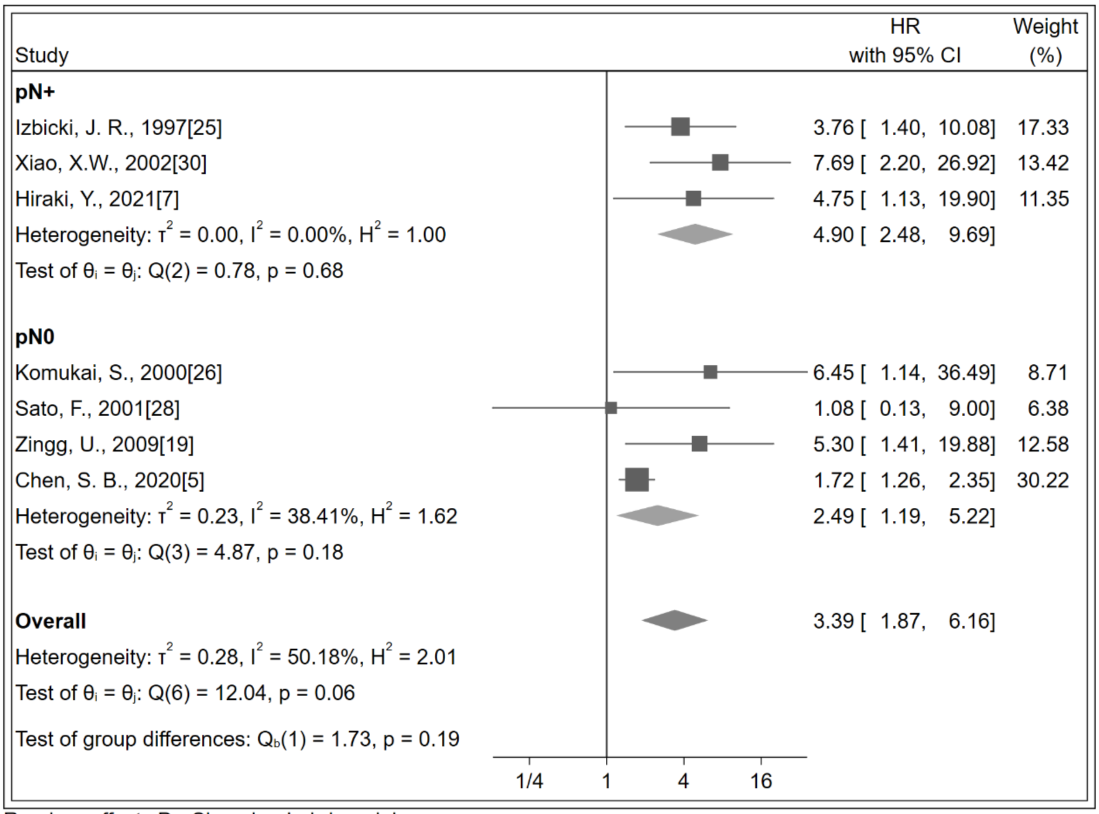

Random-effects DerSimonian-Laird model

Supplementary Appendices 12 Forest plot of region subgroup of RFS

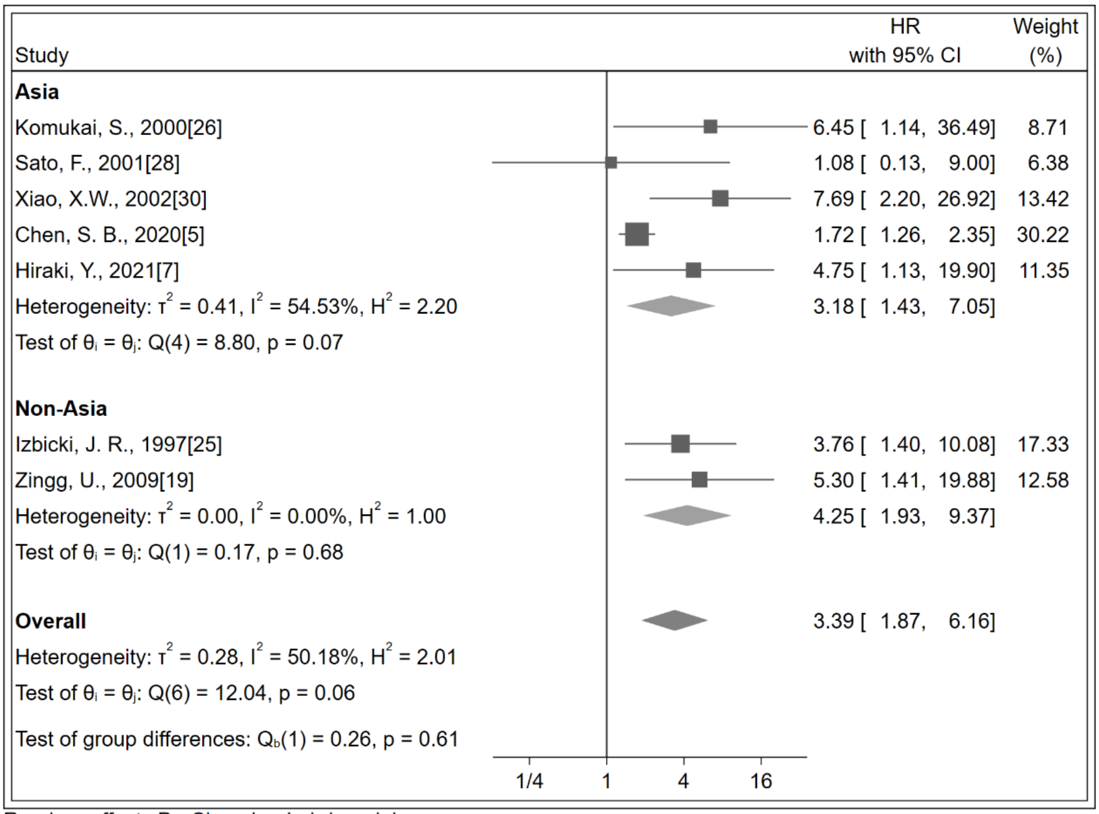

Random-effects DerSimonian-Laird model

Supplementary Appendices 13 Forest plot of study design subgroup of RFS

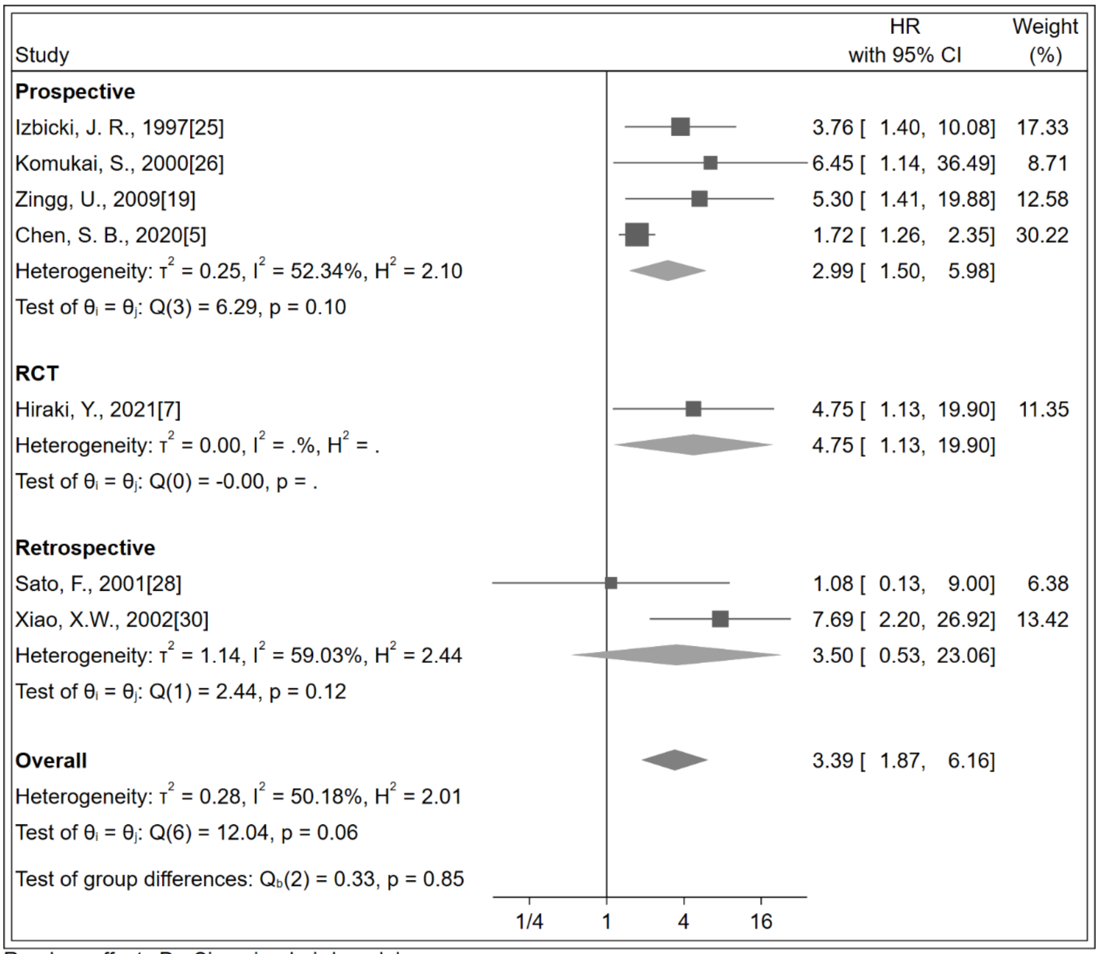

Random-effects DerSimonian-Laird model

Supplementary Appendices 14 Forest plot of antibody type subgroup of RFS

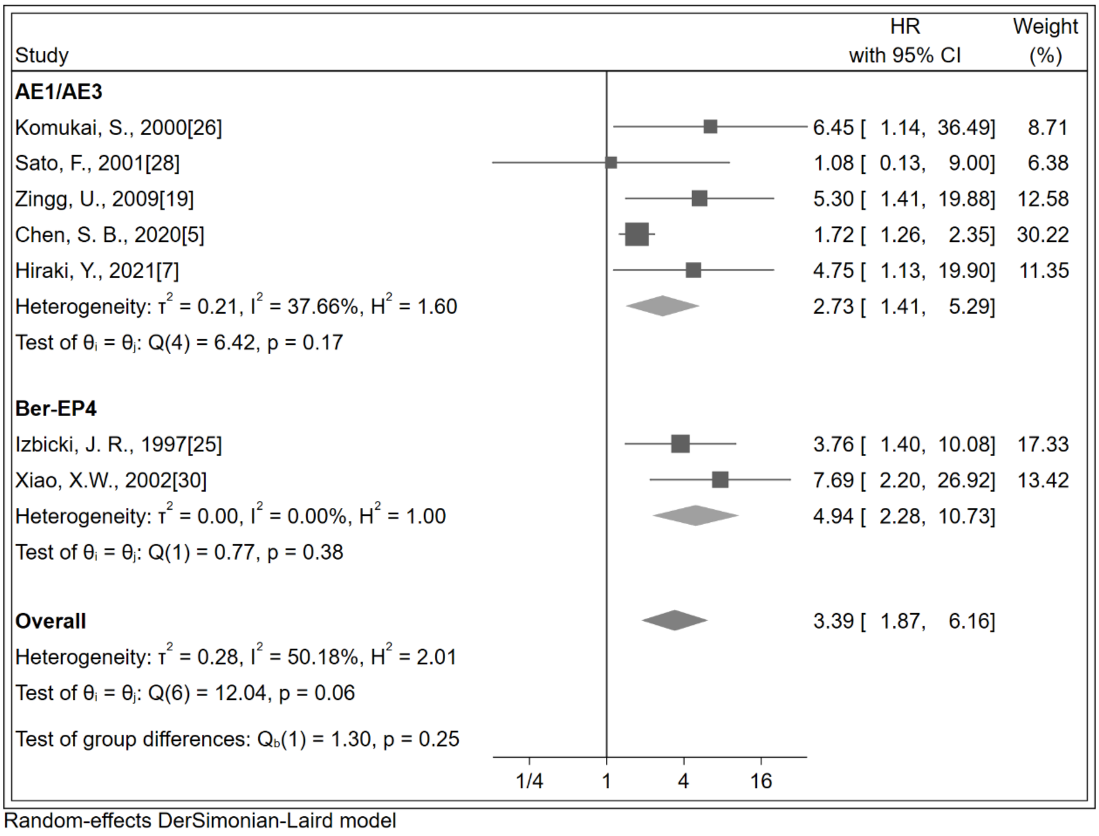

Supplementary Appendices 15 Forest plot of follow-up duration subgroup of RFS

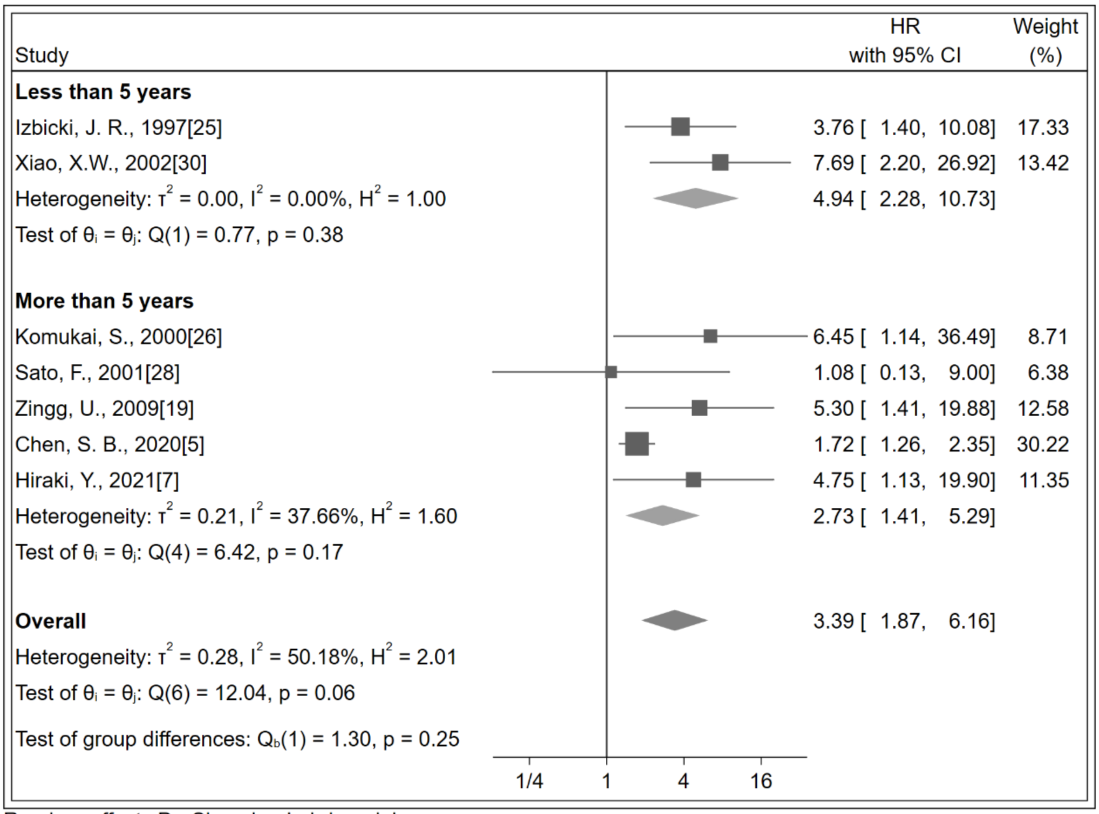

Random-effects DerSimonian-Laird model

Supplementary Appendices 16 Forest plot of single-center/multicenter subgroup of RFS

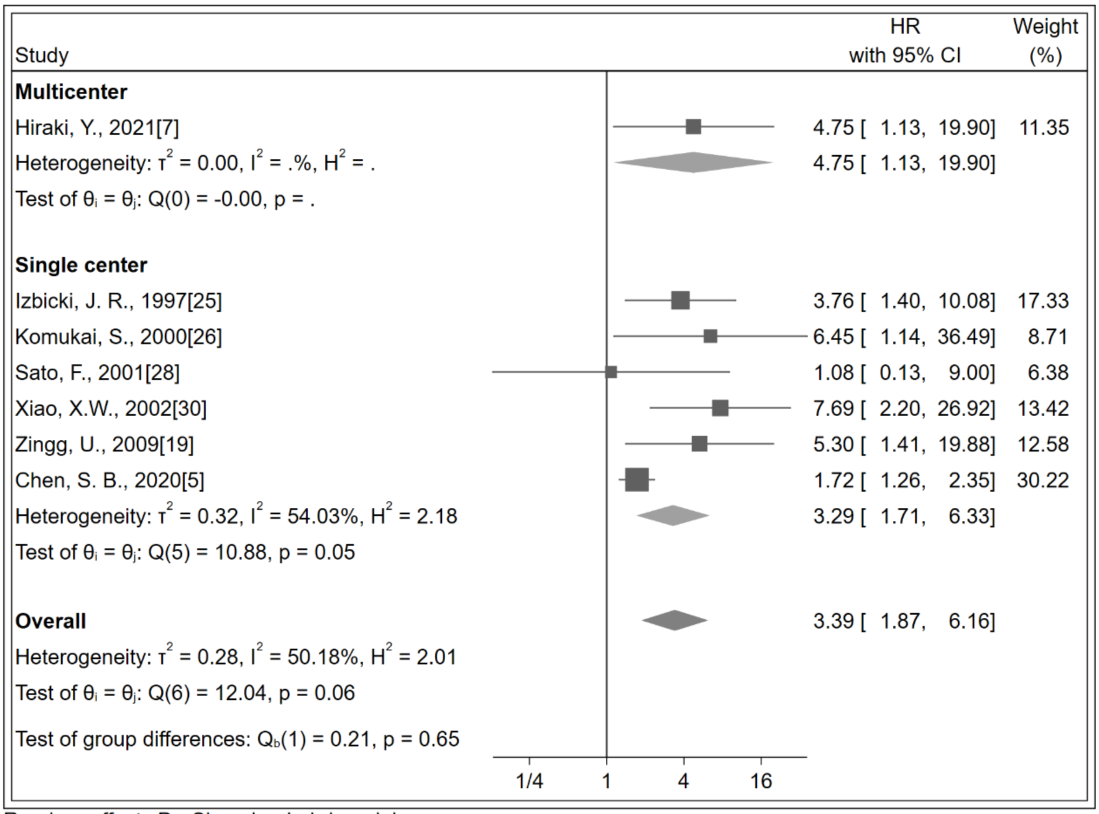

Random-effects DerSimonian-Laird model

Supplementary Appendices 17 Forest plot of tumor type subgroup of RFS

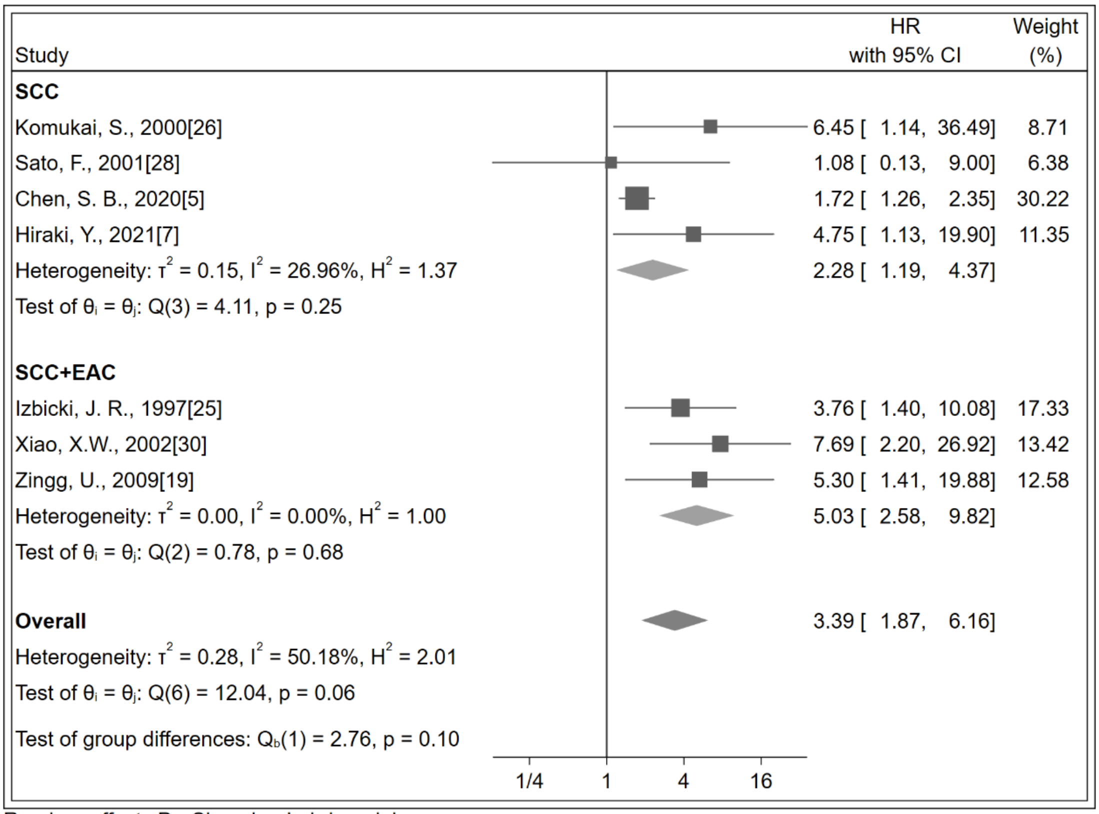

Random-effects DerSimonian-Laird model

Supplementary Appendices 18 Forest plot of univariate/multivariate analysis subgroup of RFS

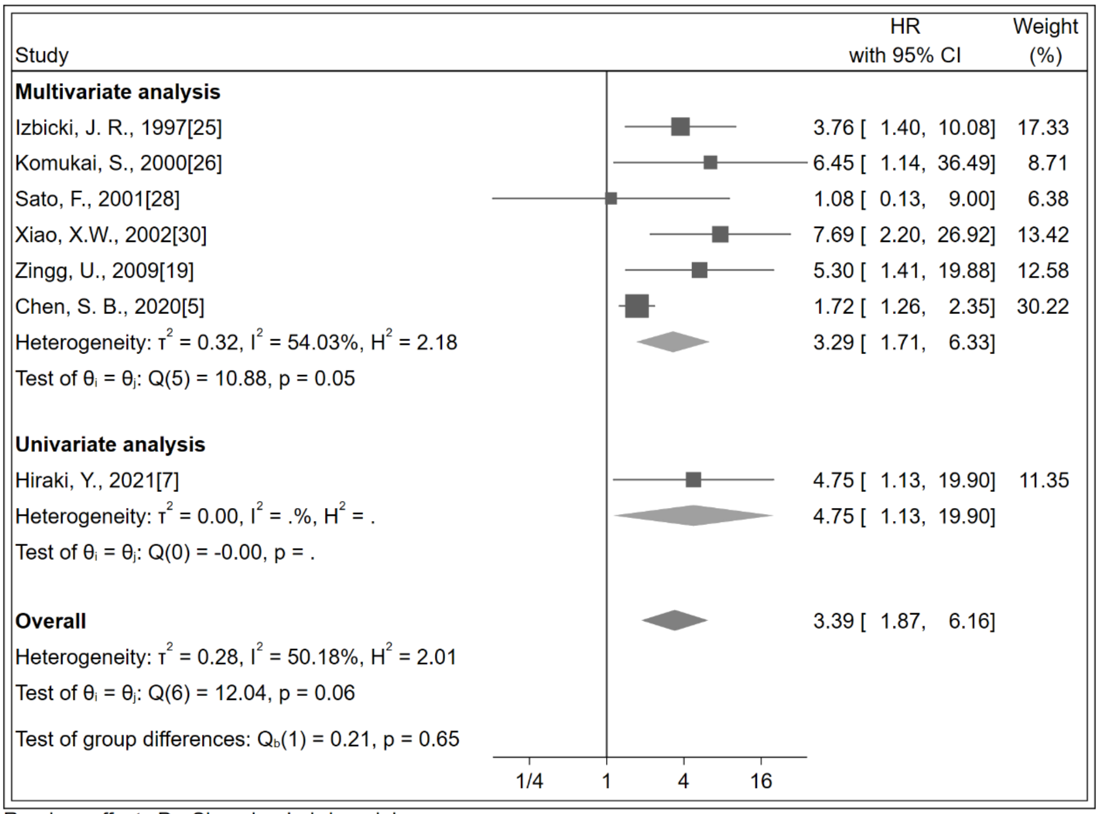

Random-effects DerSimonian-Laird model

Supplementary Appendices 19 Forest plot of pN status subgroup of RFS

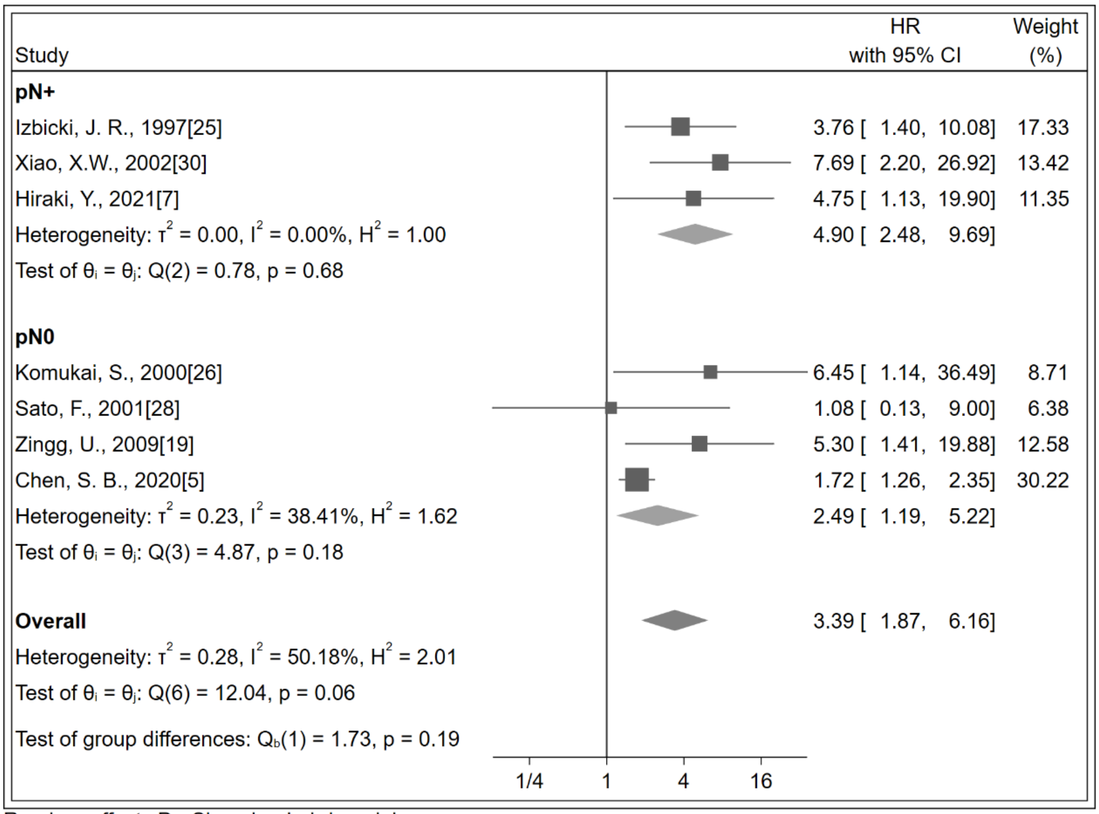

Random-effects DerSimonian-Laird model

Supplementary Appendices 20 Forest plot of follow-up duration subgroup of recurrence

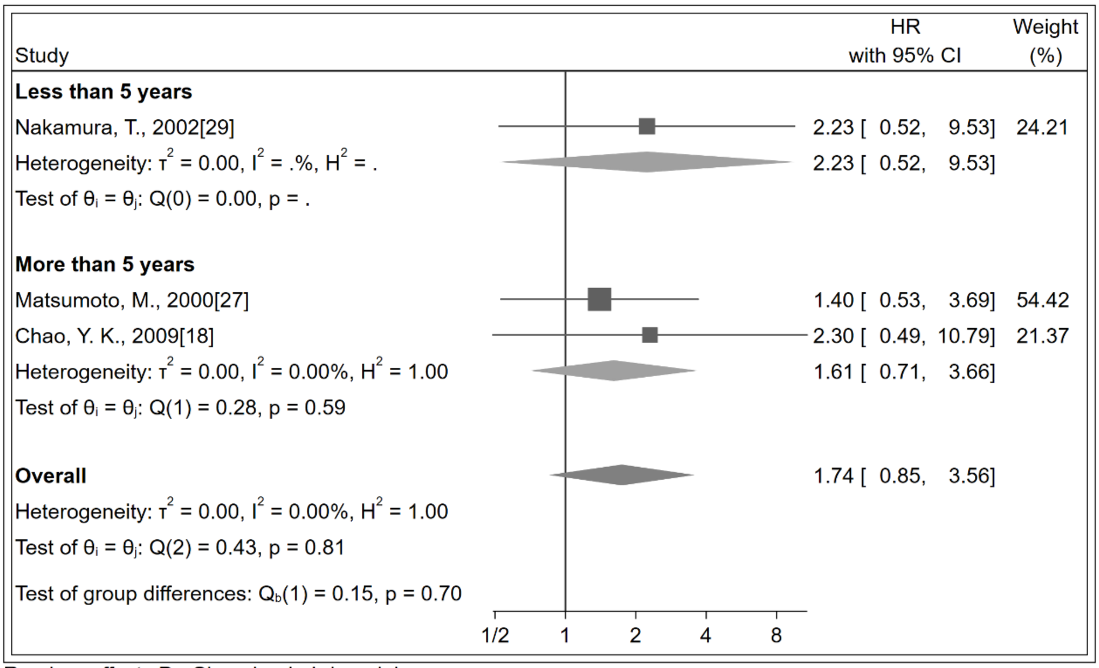

Random-effects DerSimonian-Laird model

Supplementary Appendices 21 Sensitivity analysis of OS

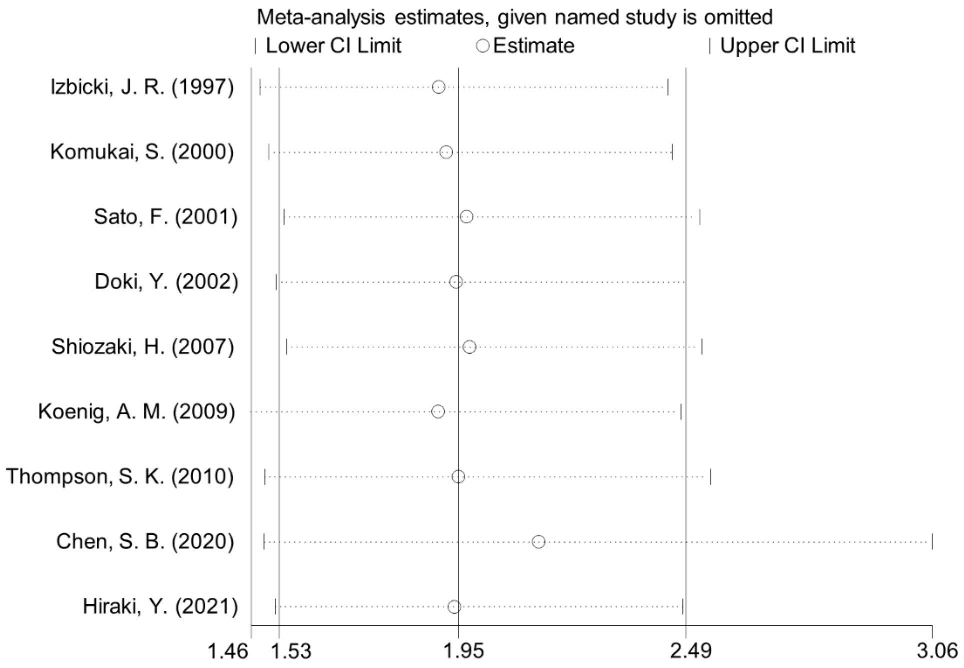

Supplementary Appendices 22 Sensitivity analysis of RFS

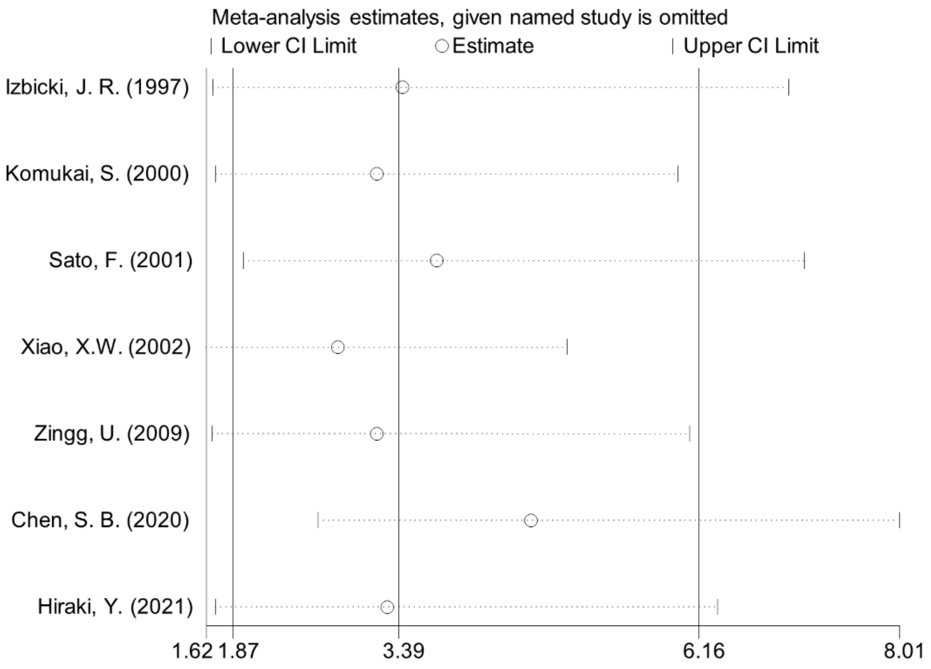

Supplementary Appendices 23 Sensitivity analysis of recurrence

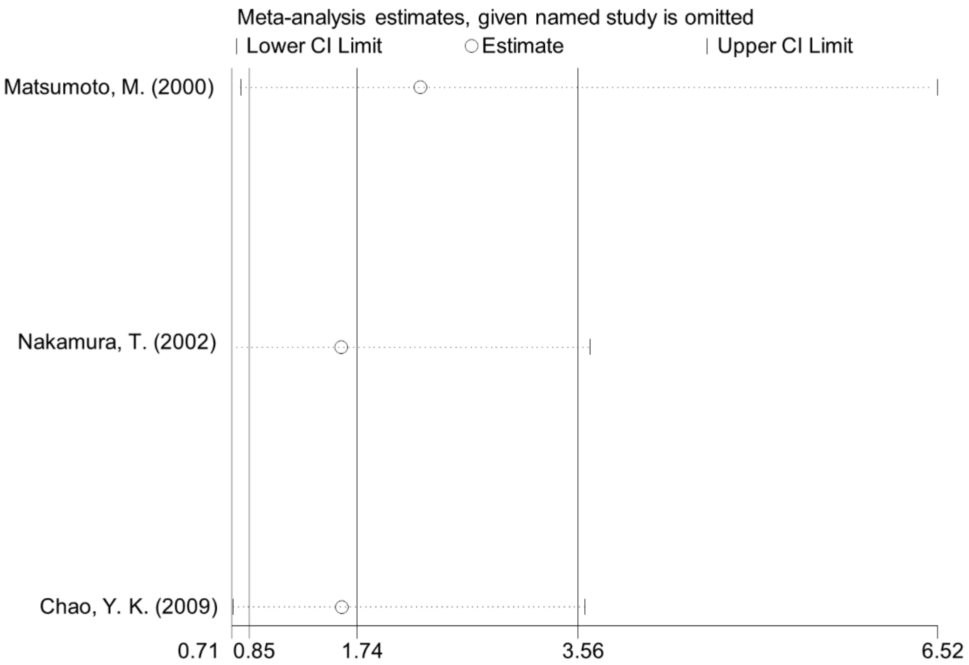

Supplement: Supplementary file 1 [file DataSheet_1.pdf]
